# Single-implant overdentures retained by the Novaloc attachment system: study protocol for a mixed-methods randomized cross-over trial

**DOI:** 10.1186/s13063-018-2606-7

**Published:** 2018-04-23

**Authors:** Raphael F. de Souza, Christophe Bedos, Shahrokh Esfandiari, Nicholas M. Makhoul, Didem Dagdeviren, Samer Abi Nader, Areej A. Jabbar, Jocelyne S. Feine

**Affiliations:** 1 Division of Oral Health and Society, Faculty of Dentistry, McGill University, 2001 McGill College Avenue, Suite 500, Montréal, QC H3A 1G1 Canada; 2 Division of Oral & Maxillofacial Surgery, Faculty of Dentistry, McGill University, Montreal, QC Canada; 3 Division of Oral Diagnostic Sciences, Faculty of Dentistry, McGill University, Montreal, QC Canada; 4 Division of Restorative Dentistry, Faculty of Dentistry, McGill University, Montreal, QC Canada

**Keywords:** Complete denture, Costs and cost analysis, Cross-over studies, Dental care for aged, Edentulous mouth, Implant-supported dental prosthesis, Minimally invasive surgical procedures, Patient outcome assessment, Patient satisfaction, Removable prosthodontics

## Abstract

**Background:**

Overdentures retained by a single implant in the midline have arisen as a minimal implant treatment for edentulous mandibles. The success of this treatment depends on the performance of a single stud attachment that is susceptible to wear-related retention loss. Recently developed biomaterials used in attachments may result in better performance of the overdentures, offering minimal retention loss and greater patient satisfaction. These biomaterials include resistant polymeric matrixes and amorphous diamond-like carbon applied on metallic components. The objective of this explanatory mixed-methods study is to compare Novaloc, a novel attachment system with such characteristics, to a traditional alternative for single implants in the mandible of edentate elderly patients.

**Methods/design:**

We will carry out a randomized cross-over clinical trial comparing Novaloc attachments to Locators for single-implant mandibular overdentures in edentate elderly individuals. Participants will be followed for three months with each attachment type; patient-based, clinical, and economic outcomes will be gathered. A sample of 26 participants is estimated to be required to detect clinically relevant differences in terms of the primary outcome (patient ratings of general satisfaction). Participants will choose which attachment they wish to keep, then be interviewed about their experiences and preferences with a single implant prosthesis and with the two attachments. Data from the quantitative and qualitative assessments will be integrated through a mixed-methods explanatory strategy. A last quantitative assessment will take place after 12 months with the preferred attachment; this latter assessment will enable measurement of the attachments’ long-term wear and maintenance requirements.

**Discussion:**

Our results will lead to evidence-based recommendations regarding these systems, guiding providers and patients when making decisions on which attachment systems and implant numbers will be most appropriate for individual cases. The recommendation of a specific attachment for elderly edentulous patients may combine positive outcomes from patient perspectives with low cost, good maintenance, and minimal invasiveness.

**Trial registration:**

ClinicalTrials.gov, NCT03126942. Registered on 13 April 2017.

**Electronic supplementary material:**

The online version of this article (10.1186/s13063-018-2606-7) contains supplementary material, which is available to authorized users.

## Background

Complete tooth loss or edentulism is a debilitating and irreversible condition that represents the ultimate consequence of oral disease [1]. Although a modest decline in the prevalence of this condition was reported for some European countries, there are still large numbers of edentulous individuals worldwide [2]. The prevalence is higher in elderly populations and can be expected to remain high for several decades [3]. Edentulism is associated with greater disability and earlier mortality in the elderly, even after adjusting for confounders such as socioeconomic status and health behavior [4]. The absence of teeth also poses a major predicament for wellbeing, as it has considerable negative impact on quality of life. Poorer oral function is closely associated with lower self-esteem and psychosocial discomfort [5].

The major purpose of dental prostheses is to reduce masticatory impairment and poorer quality of life by replacing the lost teeth. The most common prostheses for edentulism are complete dentures, which cannot completely restore lost function, e.g. chewing performance is only 30% of that for dentate individuals [6]. Many complete denture wearers are functionally impaired and, consequently, have considerable psychosocial discomfort. Such issues are mostly associated with mandibular dentures, making the mandible the primary target for dental implants [7]. Many clinical studies highlight that the mandibular implant-retained overdenture is a cost-effective choice for edentulous individuals, resulting in better patient satisfaction and oral health-related quality of life compared to conventional dentures [8]. International consensus statements have recommended the use of two implants in the mandible as the standard of care for edentulism due to favorable results and lower costs than most implant-based treatment methods [9, 10]. However, this treatment may either not be viable in some cases due anatomic or physiological conditions that are common in elderly patients or may be unaffordable for potential recipients.

The retention of a complete dental prosthesis by a single implant placed in the midline has arisen as a minimal implant-based treatment modality for the edentulous mandible. Single implant overdentures present potential advantages that may lead to their choice for certain patients. These advantages include reduced costs involved with the provision of dental care. This treatment can be especially advantageous for patients with low functional demands, such as the elderly [11]. Other benefits for this specific population include minimum operative time and low morbidity. This latter aspect seems strongly correlated to the number of installed fixtures rather than their dimensions, at least when considering the anterior edentulous mandible [12].

Outcomes of single implant overdentures are favorable considering patient satisfaction [13, 14] and oral health-related quality of life [15, 16]. These measures are substantially increased after loading a single mandibular implant in conventional denture wearers. When compared to two-implant overdentures, differences in terms of patient satisfaction and oral health-related quality of life were reported to be insignificant [13, 16]. In addition, a recent systematic review revealed no significant between-treatment differences in terms of implant survival [17]. However, treatment success rates tend to improve when avoiding immediate loading [16] and machined implants [14].

From the biomechanical standpoint, the use of a single implant is not associated with poorer fixture behavior. Maeda et al. [18] observed a favorable incidence of forces on fixtures when comparing single implant-retained mandibular overdentures to a two-implant retained prosthesis. This was found for both dome-shaped magnets and O-ball attachments. A finite element method simulation of mandibular overdentures suggests that single implants undergo favorable strain compared to numbers in the range of 2–4, without harmful loading on abutments, peri-implant bone, or the edentulous ridge [19]. Furthermore, the placement of an implant in the midline does not appear to increase the incidence of denture fractures when compared to two-implant overdentures [20].

Most studies on single-implant overdentures have used stud attachments, such as O-balls or cylindrical patrices [13–16, 21]. These systems tend to considerably improve the performance of mandibular dentures, but require regular maintenance, including change or activation of matrices. Although the incidence of matrix maintenance for single implant overdentures is comparable to that of two-implant alternatives, more than half of patients may need some reactivation, tightening, or reattachment of retentive components over a single year [13]. Such maintenance needs vary considerably among attachment systems and can be minimized by using large O-balls, e.g. 5.9-mm-wide patrices [21]. Nevertheless, this may be clinically inconvenient due to the reduced bulk of denture bases following attachment insertion and, therefore, higher risk of fracture [13].

Alternatives to well-known attachment systems may result in lower maintenance needs and other favorable characteristics. One of those alternatives is a new type of attachment dubbed Novaloc, based on mechanical retention from a polyetheretherketone (PEEK) matrix on a cylindrical patrix, that may be more resistant to wear than the nylon used in other systems. The abutments also receive an amorphous diamond-like carbon surface coating that minimizes roughness and is intended to enhance resistance of the attachment components.

In spite of the potential advantages of the Novaloc system for single implant overdentures, no previous trial has evaluated this system’s performance. A search in PubMed for the terms [overdenture* AND (PEEK OR polyetheretherketone OR “Polyether ether ketone” OR “poly-ether-ether-ketone”)] revealed a single report on bar-clip systems (1 December 2017). This suggests that there are no published clinical studies on Novaloc or any other stud system based on PEEK matrices or carbon-coated patrices. An in vitro study, however, shows promising results regarding the long-term retention of PEEK matrices compared to the traditional Locator system [22].

In this mixed-methods randomized cross-over trial (RCT), we aim to produce evidence of the efficacy of using the Novaloc attachment system to retain single-implant mandibular overdentures, compared to a traditional cylindrical attachment (Locator), for edentate elder patients. The primary outcome of this study is ratings of general satisfaction on visual analog scale (VAS) measures following three months of use with each attachment type. Through qualitative interviews, we will also describe what characteristics of these systems are considered important to patients when stating their satisfaction and preferences; we will use this rich descriptive data to help explain the quantitative RCT results.

Secondary outcomes include: (1) specific domains of satisfaction (e.g. retention/stability and hygiene); (2) oral health-related quality of life (OHIP-EDENT); (3) economic variables; (4) perceived rotation of the mandibular overdenture; (5) clinician-based outcomes, i.e. success rate of implants/overdentures and clinical complications/maintenance needs; and (6) choice of attachment.

Participants will keep their chosen attachments for another 12-month period and we will evaluate the same outcomes.

### Study hypothesis

The primary null hypothesis of the trial is that there is no difference in ratings of patient satisfaction for Novaloc and Locator attachments.

## Methods

### Design

This study is a mixed-methods, superiority RCT in elderly edentulous patients who will use two different attachment types after receiving a single implant in the mandibular midline. The Novaloc system will be compared to the commonly used Locator system as an active comparator. The performance of the mandibular overdentures will be evaluated after two consecutive three-month periods, corresponding to the two systems, totaling six months. We are not including a washout period for three reasons, with “1” and “2” supporting the unlikelihood of a carryover effect:

1. the follow-up period is long enough to elicit different responses in denture wearers’ self-reported outcomes (compatible with current treatment) [23];
2. the clinical nature of the tested devices, i.e. unlikely need for post-insertion adjustments;
3. ethical-related issues (to minimize the number of appointments for elderly participants).

**Fig. 1:**
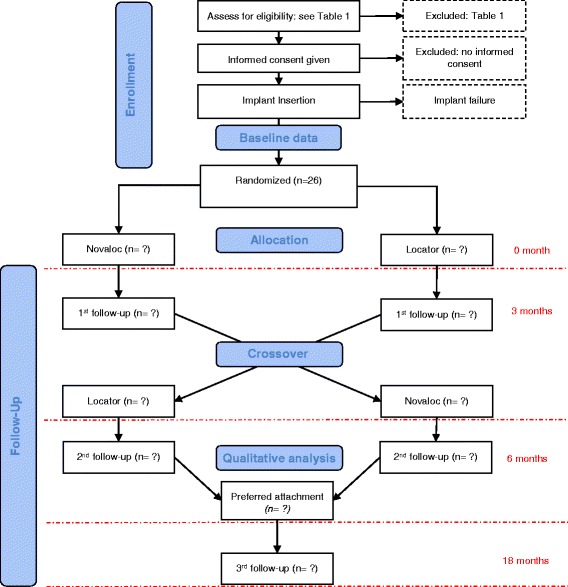
*Flowchart* of the RCT (adapted from the CONSORT statement). For each follow-up, numbers of withdrawn and lost participants will be reported with reasons

Qualitative evaluation through face-to-face interviews will follow the quantitative assessment and the results will be integrated with those of the RCT using an explanatory mixed-methods strategy. Participants will keep their preferred attachments and will be quantitatively evaluated after a long-term follow-up (12 months). Figure 1 illustrates the study flow diagram.

**Fig. 2:**
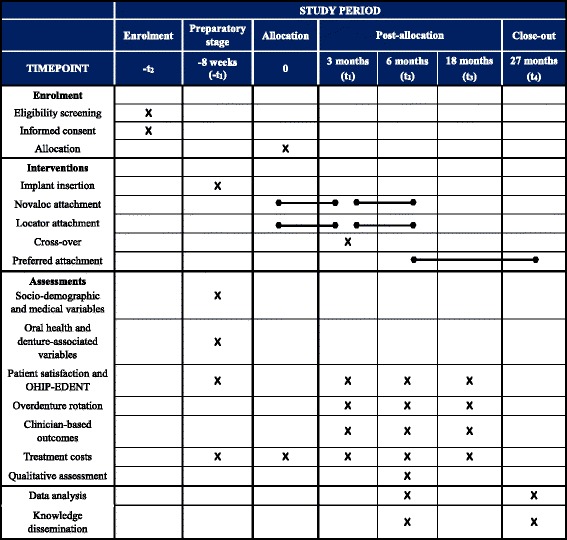
Study schedule: enrolment, allocation, interventions, baseline, and post-intervention assessments

This report was prepared according to the Standard Protocol Items: Recommendations for Interventional Trials (SPIRIT) guidelines. Figure 2 shows the standard protocol items diagram recommended by SPIRIT. Additional file 1 presents the SPIRIT checklist for this study.

### Setting and location

This trial will be conducted at two specific sites in Montréal, Canada. Most stages, including pre-screening, prosthetic care, clinical maintenance, data collection/analysis, and trial coordination, will occur in the dental clinics in the Faculty of Dentistry, McGill University. Screening, surgical procedures, and short-term postoperative care will be performed in the Department of Dentistry and Oral and Maxillofacial Surgery at the Montreal General Hospital/McGill University Health Centre (MUHC).

### Eligibility criteria

Potential participants must fulfill the inclusion and exclusion criteria detailed in Table 1. Those participants who meet the initial inclusion/exclusion criteria will undergo cone beam computerized tomographic (CBCT) scans and further exclusion criteria will be applied.

**Table 1:** Inclusion and exclusion criteria

Inclusion criteria
I. Be completely edentulous and aged 65 years or more;
II. Not have had a tooth extraction within the past six months;
III. Request implant stabilization of a mandibular conventional complete denture;
IV. Have clinically acceptable maxillary and mandibular complete dentures. Individuals without clinically acceptable dentures will be referred to receive prosthodontic care before inclusion. In any case, dentures shall present acceptable base extension and fit, maxillo-mandibular relationships, tooth wear, and aesthetics, as evaluated by a prosthodontist;
V. Have adequate bone in the anterior mandible for the placement of a single 3.3-mm-wide implant in the midline;
VI. Be able to maintain adequate oral hygiene and clean dentures;
VII. Present no systemic conditions for which minor oral surgery would be counter-indicated, e.g. severe cardiovascular diseases or uncontrolled type 2 diabetes mellitus;
VIII. Have an adequate understanding of written and spoken English or French;
IX. Be capable of giving written informed consent.
Exclusion criteria
Clinical criteria:
I. Severe/serious illness that requires frequent hospitalization;
II. Impaired cognitive function;
III. Unable to return for evaluations/study recalls;
IV. Have a history of radiation therapy to the orofacial region;
V. Have specific conditions that may jeopardize the treatment, such as alcoholism or smoking (> 10 cigarettes/day);
VI. Have acute or chronic symptoms of parafunctional or temporomandibular disorders;
VII. Previous dental implant treatment.
Radiographic criteria:
I. Any area suggestive of bony pathologic lesions;
II. Lack of minimum vertical mandibular bone height of 11 mm in the symphyseal region or width for planned implants;
III. Evident endosseous vascular structures in the planned implant site as described by Kalpidis and Setayesh [52];
IV. Mandibular ridges graded as I or II according to Cawood and Howell [28].

Inclusion and exclusion information regarding eligibility will be recorded on the recruitment form, so that the characteristics of excluded individuals can be described.

### Participant recruitment

Participants will be recruited from the Greater Montreal area using two different strategies. A first recruitment method will involve advertisements in newspapers for seniors that have been demonstrated to offer a very favorable cost/participant in a previous trial on elderly overdenture wearers [24]. Similar advertisements will be sent to dentists who teach at McGill University. The latter will be asked to refer to us any patient they consider potentially eligible for this study. Participants will be also invited to refer friends and family.

The advertisements will contain a brief study summary in lay language with contact information for the trial coordinator, including a telephone number with a voicemail and e-mail address. The coordinator will provide general information regarding the study and main inclusion criteria and will invite potential eligible individuals to a screening session. During that session, potential participants will be further informed about study characteristics, potential benefits, and risks using a study brochure and a dental model of a single implant overdenture.

### Planned interventions

#### Common implant insertion procedures

All participants will receive a single 3.3-mm-wide implant composed of TiZr alloy (Roxolid Standard Tissue Level implant, Straumann) with a minimum length of 10 mm in their mandibular midline region. Implant angulation and specific labial-lingual position will be determined by consensus with the oral surgeon, radiologist, and prosthodontist. This will follow clinical and radiographic parameters, based on the observation of soft tissues and palpation of the edentulous ridge, including an even labio-lingual distribution with minimal involvement of cortical bone and central distribution in denture bases, with at least 1 mm of resin around attachment housings whenever possible.

Implant insertion will follow the protocol defined by the manufacturer [25]. A closure cap will be placed before suturing and mandibular dentures will be hollowed above metallic components and incised mucosa. Participants will also receive Ibuprofen 400–800 mg q.i.d. for two days, then p.r.n. in case of postoperative pain. Acetaminophen 500–1000 mg q.i.d. or Naproxen Sodium 375–550 mg b.i.d. may be used as alternative medication. A second appointment will be scheduled seven days later to allow for postsurgical care, suture removal, and chairside relining with a soft material.

#### Attachment systems

After eight weeks, participants will receive their attachment abutments and matrix components will be fitted into mandibular dentures. At this point participants will receive one of the following attachment systems:

- A: Novaloc (Valoc, Möhlin, Switzerland), a novel model composed by a PEEK capsule and carbon-coated abutment. The yellow (medium) retentive component will be used in this study.
- B: Locator system (Zest Anchors, Inc., USA) with pink (medium) retentive components. Selected abutments shall have their external margins 1 mm above the mucosa.

For both attachment systems, dentures will be tested intra-orally to confirm correct seating in centric occlusion and additional room to accommodate the attachment matrix parts will be created as necessary. A spacer will be placed between the cervical part of the implant and matrix as recommended by manufacturers. When the denture is seated passively on the mandibular ridge, the prepared space will receive chemically activated acrylic resin and the dentures will again be positioned in centric occlusion. The overdenture will be removed following polymerization, inspected for any defect, corrected if necessary, and finished. Occlusal contacts will be also checked. Instructions for cleaning and maintenance will be provided to participants before denture delivery.

Each participant will receive one type of attachment randomly and wear it for three months. The attachment will then be changed and the new one worn for another period of three months. Then, each participant will be asked to choose the attachment system s/he wishes to keep.

### Randomization, allocation, and blinding

The sequence in which each participant will receive the two attachments will be decided by a list of random, computer-generated codes. Sequences will be decided based on the initial system used (ratio: 1:1): (1) A-B; (2) B-A. These codes will be secured in opaque sealed envelopes and one will be opened for each participant immediately before attachment insertion. A researcher not involved with the trial will prepare these codes and envelopes, following a simple randomization method stratified by ridge morphology (favorable - Cawood and Howell’s class III vs unfavorable/others). In spite of eventual imbalances in the number of participants per sequence, simple randomization can preclude prediction of allocation by researchers [26].

Although it will not be possible to blind participants and care providers to the treatments, researchers unaware of the participants’ allocations will conduct the outcomes assessment whenever applicable (blind assessor 1: patient-reported outcomes; blind assessor 2: cost data; refer to “Study Outcomes” below for details). We will also avoid mentioning to participants any expectation regarding performance of one system over the other. Appointments will be organized in such a way as to minimize communication between participants and to prevent potential contamination. We will request that participants keep their attachments confidential and not discuss them with the research member involved in the outcome assessment.

To confirm blinding effectiveness, participants will answer which attachment they think they have (experimental or traditional) at the end of the six-month follow-up and their conviction about the strength of their response from 0 to 10 (“not at all certain” and “extremely certain,” respectively) [27].

### Study outcomes

All patient-reported outcomes, except for prosthesis rotation, will be collected at baseline, together with sociodemographic data, including age, gender, level of education, and income. Clinical data will be also collected before treatment, such as ridge morphology [28], amount of time edentulous, and age of existing dentures.

Participants will return for outcome assessment after three months using each attachment, for a total follow-up time of six months. A three-month period is enough to observe a significant decrease in the retention force of Locators [29] but is of unlikely length for adverse events associated with other components. Moreover, a slightly longer period, i.e. six months, results in similar patient satisfaction with mandibular overdentures [30]. Participants will be evaluated again 12 months following the cross-over trial for an appraisal of maintenance needs/adverse events. A 12-month period will be enough to reveal attachment maintenance events for most participants with Locators [21], as well as some uncommon events, e.g. denture base fractures [13].

In each assessment session, participants will provide data on:

1. Satisfaction with overdentures. The McGill VAS Satisfaction questionnaire [31] will be used to measure general satisfaction (primary outcome), as well as satisfaction with other functional aspects of the prostheses using 100 mm VAS. Other aspects include ability to chew, comfort, stability, aesthetics, ability to speak, and ability to clean. Some of the 13 items in the questionnaire include ability to chew specific foods (lettuce, soft bread, hard cheese, steak, raw apple, and raw carrot). These were selected because they represented different textures and hardnesses and were ranked by a group of complete denture patients from “very easy” to “very difficult” to chew. Patients will rate their ability to chew these foods on a 100 mm VAS with anchors of “not at all difficult to chew” to “impossible to chew.” Patients will be trained to use this type of scale before answering the questionnaires. Previous studies have shown good properties for the McGill VAS Satisfaction questionnaire. Besides good internal consistency and reproducibility [32], its ability to discriminate between different clinical conditions denotes good construct validity [30, 33].
2. Oral health-related quality of life. Participants will be asked to complete the Oral Health Impact Profile for edentulous people (OHIP-EDENT), which is an abbreviated version of the original 49-item OHIP that was tested specifically with edentulous populations [34]. This questionnaire is composed of 20 items that can be grouped into subscales, representing different domains/dimensions of perceived impact, such as functional limitation or social disability. This abbreviated version shows good reliability and discriminant validity, similar to the original OHIP [34]. Although seven subscales were originally purported [35], we will use a four-domain model as recommended by recent factor analysis studies [36, 37].
3. Overdenture rotation. Patient perception of rotation of the mandibular overdenture will be assessed by two questions, as used by Kimoto et al. [38]: (1) Does your denture lift at the back when you chew? (yes-no); (2) How much does the lifting of your denture bother you? (100 mm VAS).
4. Clinician-based outcomes will be assessed for each three-month period. Participants will be evaluated based on a standardized clinical form, which will be completed immediately following each appointment. Overdentures will be classified as successful if they can function in the way that they were intended, with additional retention provided by the attachment system. For the determination of implant success and survival, the implants will be classified at each data collection appointment according to Misch et al. [39]. Clinical evaluation will also include the following parameters [30]: (a) presence of plaque; (b) presence of calculus; (c) depth of peri-implant pockets; (d) bleeding on probing; (e) signs of swelling or inflammation. The presence of suppuration and tenderness will also be noted. Probing depth will be measured at four sites for each implant (mesially, labially, distally, and lingually) using a periodontal probe.
5. Treatment costs. We intend to gather data on both direct and indirect costs to compare the differences in cost-effectiveness between the two attachments systems. The effectiveness is measured by any difference in overall patient satisfaction (primary outcome) at baseline and at the three- and six-month follow-up visits. The size of the effect may vary at each of the two intervals. This analysis is based on the following assumptions: (1) the total cost at the end of the 6-month evaluation period would be the same between sequences 1 and 2; (2) the implant placements are deemed clinically successful at given intervals (three and six months post treatment); (3) no surgical or prosthetic complications are encountered during the six-month post-treatment period. Cost-effectiveness between the two attachments systems will be determined by calculating incremental cost-effectiveness ratios (ICER), based on baseline–post-intervention differences in satisfaction.

### Sample size estimation

Sample size for the quantitative phase was determined based on the primary outcome of this study (patient satisfaction). According to previous trials, a minimally important difference of 10 mm was considered for the estimation [40], as well as a standard deviation of the difference in satisfaction of 7.5 mm [41]. A two-sided alpha of 0.01 will be also considered to compensate for the number of secondary outcomes. Based on a power of 90%, this trial requires at least 21 participants [42]. An additional 20% will be added to the planned sample to compensate for possible dropouts, thus resulting in a total of 26 participants.

### Statistical analysis

All quantitative data will be entered and analyzed in a blinded fashion. Such data will undergo descriptive analysis and will be transformed in cases of considerable deviation from normality. Clinical data will generate a composite variable representing clinical adversities; both the compound measure and separate event types will be analyzed.

Mixed linear models will be used to test the effect of interventions and follow-up time (three vs six months), as well their interaction.

The primary outcome will be further tested for carryover effects by fitting another mixed linear model with the two treatment periods and testing for the intervention by period interaction. Other approaches to assess the primary outcome will involve the inclusion of the randomization strata (ridge shape), age, baseline results, and perceived denture base rotation as covariates in separate models, one covariate each time. Analysis for carryover will be performed for cost estimates.

Results will be evaluated according to the intention-to-treat principle. In the case of unbalanced missing data among interventions or loss of 5% (*n* > 1) or more of participants, different strategies will be attempted for imputing primary outcome data, as recommended by Dziura et al. [43]; multiple imputation will be used for patient satisfaction based on least squares regression with at least five datasets. In the case of “missing not at random” (MNAR) data, we will repeat analyses after a baseline-observation-carried-forward approach (i.e. withdrawn participants will be considered as dissatisfied as before receiving implants). A second analysis will be performed with imputed values and cross-checked. Statistical tests will be performed using SPSS 21.0, considering α = 0.05.

### Qualitative assessment

A qualitative assessment will follow the quantitative phase to better understand any emergent themes in addition to the following: (1) patients’ reasons for choosing one attachment system; (2) their perceptions of the advantages and disadvantages of each attachment; and (3) their experience of living with a single implant to retain their mandibular overdentures. We will adopt a descriptive approach [44], conducting individual semi-structured interviews, that constitutes a powerful method to obtain an in-depth understanding of patients’ experiences and preferences [45].

We will conduct individual semi-structured interviews with all study participants (n = 26). The interviews will be held outside of the clinics in a place conducive for a confidential discussion. An experienced research associate will use an interview guide that describes the interview procedure and contains open-ended questions on the abovementioned themes. Since this is an explanatory mixed-methods design, we will follow appropriate guidelines, in that the interview guide will be developed based on the results of the quantitative outcomes and on which results require further explanation or clarification. Participants will be encouraged to express themselves spontaneously in a conversational manner. Each interview (about 1 h long) will be digitally recorded and transcribed; the transcription will serve as the basis for qualitative data analysis.

The analyses will consist of several stages: (1) summarizing each interview using a contact summary sheet; (2) coding the transcribed interviews; and (3) summarizing and interpreting the data. The contact summary sheet, as recommended by Miles et al. [46], will be completed immediately following each interview and provide a preliminary assessment of important findings. Coding of the transcribed interviews under each theme will be done with N’Vivo software (QSR). To improve coding quality, the research associate and one of the researchers will undertake to compare two transcripts chosen at random: each will code these accounts independently and then meet to compare results and to revise the codes when necessary. The research associate and the researchers will then identify and describe all relevant themes related to our research objectives.

### Integration of datasets

Quantitative and qualitative datasets will be compared to assess their eventual correspondence. This will be done by a follow-up of results with a joint display [47] of both sets to determine whether quantitative patient satisfaction and preferences can be explained by qualitative findings.

### Long-term follow-up

Once participants provide six-month data and choose the attachments they wish to keep, they will receive new polymeric components. Some participants might prefer to have no specific preference; in that case, we will keep the last type used. Then, we will schedule them for another appointment after 12 months, during which session the same outcome data will be gathered. The same statistical strategies will be employed for the analysis of these long-term data.

In the meantime, participants may contact the research team for unscheduled appointments. Any event, such as maintenance and clinical complications, as well as time and procedures carried out, will be reported as part of the collected outcome data.

### Data management, monitoring, and auditing

A data monitoring committee composed of two independent researchers will check collected data regularly. These researchers shall have no relationship with the trial sponsors. Moreover, the Institutional Review Board (IRB) at McGill University may conduct an independent audit at any time.

### Risks, participant safety, and trial adherence

Participants may experience the usual postoperative pain and sensitivity associated with minor oral surgical procedures, such as placing dental implants. Specific events may include pain in the surgical site (mandible or mucosa), redness and sensitivity/tenderness of oral mucosa, discomfort associated with changes in prostheses, or discomfort from local anesthesia. These events tend to be transient and localized.

We will record all adverse events observed at each follow-up and at any non-scheduled visits. Possible events include loosening or loss of implants or attachment components, problems with dentures (missing fit, fracture of bases or teeth), and some difficulties to remove and insert the mandibular denture according to a correct pathway. We expect a relatively low rate of occurrence, comparable to other studies [14, 16–18, 20].

Prosthetic adverse effects during the study will be managed as part of the research, by using standard repair or replacement of prosthetic components or dentures, or management of peri-implant tissues. In the unlikely case of a failed implant, we will offer the possibility of replacing it one time at no charge. If the participant does not wish to or cannot receive a replacement, the mandibular prosthesis will be converted to a conventional complete denture by relining or rebasing.

We expect good adherence levels due to the provision of an effective treatment modality (more stable mandibular dentures) as a major benefit combined with the need for regular maintenance recalls, which are integral to overdenture cases. We will also remind participants a few days before each follow-up appointment by telephone or e-mail, according to their preference. Each participant will be offered CAD$ 25.00 per data collection visit as compensation for their travel expenses, corresponding to each three-month follow-up (cross-over stage) and the 12-month final follow-up. A total of CAD$ 75.00 will be offered for the three appointments required for the trial.

### Confidentiality

All quantitative and qualitative data from each participant will receive an identification code and will be kept strictly confidential. Information linking the participants’ identities to the codes will be kept in a password protected file and computer.

### Dissemination and knowledge transfer

Findings will undoubtedly be relevant for clinicians involved in the prosthodontic care of the elderly and will have an impact on decisions concerning implants and attachments for this population. The relative efficacy of each attachment, as well as its economic characteristics, will determine which would be more appropriate for an accessible and minimally invasive treatment for edentulism.

Results will be presented at major scientific conferences, including the International Association for Dental Research (IADR) General Session and conferences held by the International Team for Implantology (ITI), as well as at other clinical meetings. Full-text reports will be sent for publication in major journals read by dentists, including *Clinical Oral Implants Research*, the *International Journal of Oral and Maxillofacial Implants*, the *Journal of Dental Research*, the *JDR Clinical and Translational Research*, or the *Journal of Dentistry*. Future reports from this trial will be prepared according to the to the Consolidated Standards of Reporting Trials (CONSORT) Statement, more specifically to its extensions for non-pharmacologic treatment and patient-reported outcomes [48, 49].

We will continue to follow study participants after the cross-over trial to collect long-term data. Participants will receive standard clinical care but will be also invited to provide data regarding trial outcomes yearly.

## Discussion

This RCT will provide comprehensive data regarding the clinical performance of single implant overdentures using a novel attachment system. Results will lead to better evidence-based recommendations for the treatment of edentulous patients, which have been hindered by the paucity of comparative studies involving single implant overdentures.

The benefits of one minimally invasive implant could be further enhanced if the fixtures used will minimize the need for adjunctive procedures, such as ridge augmentation. Narrower implants may result in lower costs, minimal discomfort, and/or less postoperative morbidity in cases where augmentation would be needed for standard implants [12]. Therefore, this study intends to use TiZr alloy implants that allow for improved mechanical resistance and are reported to result in favorable success rates [50].

Patients’ responses to dental prostheses are complex and can be described by a series of outcome variables covering different aspects. Therefore, we will elucidate these responses and any differences between the Novaloc system and the conventional alternative by combining the quantitative and qualitative data with clinical data. Qualitative methods can provide a detailed understanding of the experience of patients, as demonstrated by a previous study from our team on the reasons for declining treatment with implant overdentures [51]. The use of a mixed-methods approach will integrate the quantitative findings from the clinical trial through an in-depth interpretation of patient perspectives through qualitative methods [47].

We expect that the results will be of major relevance to clinicians who provide implant-assisted treatment to edentate patients. Resulting guidelines/recommendations can improve dental prosthetic care for the edentate elderly population, in which a huge proportion are unable to access more complex treatment modalities.

## Trial status

Recruiting since May 2017.

## Additional files

Additional file 1: SPIRIT 2013 Checklist. (DOC 121 kb)

Additional file 2: Funding documentation. (PDF 59 kb)

Additional file 3: Ethical approval document. (PDF 766 kb)

Additional file 4: Consent form in English. (PDF 2335 kb)

## Abbreviations

CAD$: Canadian dollars
CBCT: Cone beam computerized tomography
CONSORT: Consolidated Standards of Reporting Trials
IADR: International Association for Dental Research
ITI: International Team for Implantology
JDR: Journal of Dental Research
OHIP-EDENT: Oral Health Impact Profile for edentulous people
PEEK: Polyetheretherketone
RCT: Randomized cross-over trial
SPIRIT: Standard Protocol Items: Recommendations for Interventional Trials
VAS: Visual analog scale
