# Supplementary material for: Single-implant overdentures retained by the Novaloc attachment system: study protocol for a mixed-methods randomized cross-over trial
Source: Trials. 2018 Apr 23;19:243. doi: 10.1186/s13063-018-2606-7 (PMC5913792; doi:10.1186/s13063-018-2606-7)
Supplement: Supplementary file 2 — Funding documentation. (PDF 59 kb) [file 13063_2018_2606_MOESM2_ESM.pdf]

International Team for Implantology

Raphael de Souza DDS, MSc, PhD
McGill University
Faculty of Dentistry
Oral Health and Society
2001 McGill College Avenue, Suite 500
Montreal H3A 1G1
Canada

Basel, 28 February 2017

ITI Research Grant Application No. 1185_2016: Confirmation of ITI Research Funding, Dr. Raphael de Souza

To whom it may concern

With reference to the above application submitted to the ITI we take pleasure in confirming that the ITI's Research Committee has agreed to support the project "Single implant overdentures retained by the Novaloc attachment system: a mixed method randomized trial" to be led by Dr. Raphael de Souza with an ITI research grant to the amount of USD 99'922.82

The research project begins in April 2017 and the projected end date communicated to ITI by Dr. Raphael de Souza will be in December 2020.

The grant will be transferred to the bank named below to an account whose details were communicated to ITI Headquarters as follows:

Name of the bank:	RBC Royal Bank
Address of the bank:	1 Place Ville Marie, H3C 3A9 Montreal, Canada
Account holder:	McGill University

The grant is to cover expenses associated with the project above. The payment schedule is as follows:

1.	After provision of proof of ethical committee approval (March 2017):	USD 61'763.74
2.	After provision of the status report (October 2017):	USD 28'166.80
3.	After receipt of the final report:	USD 9'992.28 (final 10% of the grant)
	Total:	USD 99'922.82

In the case of an animal study or human study, the first installment will be transferred as soon as proof of Ethical Committee approval has been provided to ITI Headquarters.

ITI International Team for Implantology

ITI Headquarters
Peter Merian-Strasse 88
CH-4052 Basel

Tel. +41 (0)61 270 83 83
Fax +41 (0)61 270 83 84
research@iti.org
www.iti.org

A status report must be provided to the ITI Research Committee once a year and sent to ITI Headquarters at research@iti.org, and an intermediate report at the halfway point in the study, using the template that will be provided by ITI Headquarters.

A comprehensive final report must be provided at the end of the project. Ten percent of the agreed grant amount will be held back and transferred after receipt of the final report. A copy of the publication resulting from the ITI-funded project must be provided to ITI Headquarters as soon as available.

An invoice needs to be sent to ITI Headquarters by the Principal Investigator's institution on the dates above for each installment to be paid. Please refer to page 3 of this document for further information on how to issue an invoice.

Any unspent funds after completion of the project should be returned to the ITI.

The terms and conditions that apply to this grant can be found under "Terms and Conditions" of the ITI Research Grant application form submitted by Dr. Raphael de Souza when applying for the grant.

Dr. Raphael de Souza's project is registered as ITI Grant number 1185_2016 which must be quoted in all future correspondence.

With best regards

Prof. Dr. Lisa Heitz-Mayfield
Chair of the ITI Research Committee

Dr. Kati Benthaus
Executive Vice Director of the ITI

Information on how to issue an invoice:

An invoice needs to be sent to ITI Headquarters by the First Applicant's institution on the dates noted in the payment schedule above for each installment to be received.

Invoices must be made out to:
ITI Foundation
Research Grant Administration
Peter Merian Strasse 88
4052 Basel
Switzerland

Invoices must be made out:

- in English or German
- in the currency of the grant award
- on the First Applicant's[1] institution's letterhead and quote the institution's name and address

Invoices must quote:

- the ITI Grant number
- the First Applicant's institution's VAT identification number
- the ITI Foundation's Swiss VAT number: CHE-101.991.665 MWST
- the name and the address of the bank
- the name of the account holder
- the bank account number and SWIFT (non-European countries)
- IBAN and SWIFT (Europe)

Invoices are to be emailed to ITI Headquarters to research@iti.org

[1]First Applicant = individual listed as «First Applicant" on the ITI Research Grant application form submitted to the ITI
