# Supplementary material for: Single-implant overdentures retained by the Novaloc attachment system: study protocol for a mixed-methods randomized cross-over trial
Source: Trials. 2018 Apr 23;19:243. doi: 10.1186/s13063-018-2606-7 (PMC5913792; doi:10.1186/s13063-018-2606-7)
Supplement: Supplementary file 4 — Consent form in English. (PDF 2335 kb) [file 13063_2018_2606_MOESM4_ESM.pdf]

McGill
Faculty of Dentistry

# INFORMATION AND CONSENT FORM

## Single-Implant Overdentures Retained by the Novaloc Attachment System: a Mixed Methods Randomized Cross-Over Trial

**Principal investigator:**
Dr. Raphael F de Souza
Faculty of Dentistry, McGill University
2001 McGill College, suite 534, Montreal, Quebec H3A 1G1
Tel:(514) 398-4777; Email: raphael.desouza@mcgill.ca

**Research team members:** Professor Jocelyne Feine, Dr. Shahrokh Esfandiari, Dr. Nicholas Makhoul, Dr. Christophe Bedos, Dr. Samer Abi Nader, and Dr. Didem Dagdeviren
Faculty of Dentistry, McGill University

## 1. Purpose of this Consent Form

We are inviting you to participate in a research study designed to assess the success of a special type of treatment using dental implants, and to compare two types of connectors used to attach your lower denture to these implants. The results of the research will provide further knowledge about how to treat elderly patients who lose teeth. This consent form aims to:

a. inform you, as completely as possible, of the nature, purpose and risks involved in the study;

b. provide you with the necessary information you may need to decide if you will participate or not, according to your personal goals;

c. help us talk with you about your disability and its treatment.

Please read this consent form carefully and ask any questions that you may have before deciding whether or not to participate in this study. The researchers are here to help you understand completely, so please feel free to ask about anything you may want to know about the study. Please take as much time as you wish and feel free to discuss this study with your family or friends before deciding. Your participation is entirely voluntary, and if you decide not to participate, there will be no penalties or loss of benefits to which you are entitled.

If you prefer conventional dentures or are fully satisfied with your present dentures, you should NOT consider participating in this study.

## 2. Introduction

For many years, dentists have provided complete dentures for people with no teeth, but dentures only stay in the mouth by holding onto the gums and bone. Many denture wearers are able to wear an upper denture without difficulties, but have difficulties wearing their lower dentures because of looseness and discomfort. Without teeth, jawbones shrink, causing further loosening of dentures, making it hard for people to chew and speak with ease.

Studies have shown that people who wear lower dentures are significantly more satisfied with them when dental implants support them. Dental implants can make a lower denture more stable and improve chewing and comfort. Implants are shaped like dental roots, inserted in the jawbone, and dentures snap onto them.

Elderly patients are most likely to have no teeth and little remaining bone; a lack of a strong bone foundation makes it difficult to securely place implants. In addition, the cost of implant treatment is usually far beyond the means of most elderly people. Therefore, using a minimum number of implants, such as a single implant in the front part of the lower jaw where enough bone is most likely to remain, will also lower the cost. The denture can be attached to the implant using a connector that holds the denture tight.

Many dentists have considered two implants as the minimum number to securely hold a lower denture in place. We believed that a single implant could not last in the mouth for long or hold the denture strongly enough. However, recent studies show that a single implant can stay in place for the same time as with more than one implant. The satisfaction of patients with their lower dentures is similar after receiving one or two implants.

We will compare a new type of connector to another often used around the world; both are attached with a single implant in the lower jaw. Participants using these will rate their satisfaction with both types and describe their experiences with each connector in individual interviews. The costs for each type will also be recorded to determine the cost-benefit associated with each connector. The results of this study will guide dentists and patients when choosing the most appropriate connector for their needs.

## 3. Objectives

The primary goal of this study is:

- To learn about your satisfaction with a new connector type called Novaloc, which is attached to a single implant in your lower jaw, compared to a traditional type called Locator. We also wish to know which would be your preferred connector and some reasons as to why you may prefer this connector. We plan to ask you why you prefer one of these connectors in an interview.

Our secondary goals involve comparing these connectors according to:

- How they affect quality of your life and questions about how well the connectors and dentures work;
- how well your dentures will work with each connector from the dentist's viewpoint, including the need for repairs and survival of all denture parts and implants;
- estimated cost of the treatment.

## 4. Study Procedures

Our goal is to include 26 participants in this study. You may be eligible to participate in this study if you:

- Are 65 years or older and have no natural teeth for at least 6 months;
- Have a complete set of dentures in good condition, and would like the lower denture to be supported by an implant;
- Have enough bone in the top part of your lower jaw;
- Are in relatively good health and able to clean your dentures. Please tell us if you have any disease or are taking medicines (including those for thinning your blood or for weak bones).

If you are interested in participating, we will do some exams and fill out forms similarly to what is done in routine dental treatment with implants. This will include asking questions about your general and oral health, an examination of your mouth and x-rays of your jawbone. The results of these exams will be compared to a list of characteristics our study needs from participants to confirm if you are eligible to participate.

After this first appointment, you will be told whether you can participate. If you are interested in participating, we will need you to sign this consent form. You can take the consent form home to discuss with family, dentist, denturist or others, if you wish. After you sign the consent form, we will collect some of the information described in the objectives, and schedule another appointment for installing the implant.

When you come for the 2nd appointment, you will receive the implant (3.3 mm wide and at least 10 mm long). First, we will numb your lower jaw to prevent pain. Then, we will prepare the jawbone and install the implant. We will place some stiches and then the denture will be trimmed above the implant and stiches. Then, we will wait for the jawbone to heal for eight weeks or more. In the meantime, we will schedule at least one visit to see if the healing is going well, after approximately one week.

After a healing period of eight weeks, we will install components that will stabilize your lower denture. We will screw a cylindrical metal part into your new implant, whereas the lower denture will receive another part containing a plastic capsule. The plastic capsule snaps into the metal cylinder and provides extra stability. Each connector type (Novaloc or Locator) is composed of a different cylinder shape and material, and has a specific capsule, with different shape and type of plastic. **You will be given one of the two connectors based on selection by a random (by chance) selection** (we will count the beginning of the study time from this moment). We will make a small hollow in your lower denture that will serve to accommodate the component and bind it to the denture, using an acrylic material.

**Three months after** you receive the first connector type, we will schedule an appointment to collect all the information listed in the objectives (questionnaires answered by an examining dentist and you), except for the interview about your preferences. You will then receive another type of attachment (if you start with Locator, you will receive Novaloc now, and vice-versa). We will remove the old component from the denture by gently grinding away the acrylic around it.

**Three months later** (total time: 6 months), we will collect the same information gathered during the previous appointment. We may keep the same component type or place the previous one back, as you prefer. After this, another researcher will interview you outside of the clinic (estimated time: 1 hour) and ask about your preferences and experiences with the components and the treatment you received as a whole.

**One year later** (total time: 18 months), we will collect information in the clinic using the same questionnaires done for the three-month appointment.

We may also take pictures of your dentures and mouth and keep used component pieces for study material. The table below shows the stages of the study. Some minor appointments (removing stiches, taking x-rays and some eventual adjustment of your dentures) will also be necessary.

| **Visit** | **Stages of the study** | **Treatments / data collection** | **Duration (hours)** |
|---|---|---|---|

| | | | |
|---|---|---|---|
| 1 | Screening | Questionnaire, mouth exam, consent form | 1.5 |
| 2 | Implant insertion | Minor oral surgery for insertion of implant, grinding your denture above the implant | 1.5 |
| 3 | Prosthetic modification | Questionnaires, mouth exam, hollowing your denture and installing retentive components | 1.5 |
| 4 | First data collection (3 months) | Questionnaires, mouth exam, changing retentive components | 1.5 |
| 5 | Second data collection (6 months) | Questionnaires, mouth exam (we may change components if you wish), interview | 2.5 |
| 6 | Third data collection (18 months) | Questionnaires, mouth exam | 1 |

If you leave this study before or at the end, you will be referred to a dentist of your choice for occasional check-ups and regular or emergency dental care. Any unplanned change to received treatment will likely lead to your exit from the study; unplanned changes include: new dentures, changing components or more implants (please bring these changes to our attention, if they happen).

## 5. Associated risks and inconveniences

The risks associated with treatment are the same expected for minor oral surgery and dental prosthetics. Prior to implant placement, you will receive an anesthetic injection to reduce sensitivity to pain. An allergic reaction to the injection is rare, although possible. If this occurs, safe, standard procedures will be followed. These could include providing anti-histamines and / or adrenaline.

You may experience some sore spots under your dentures after the placement of implants or retentive components. If this happens, the dentures will be adjusted for you as necessary. Allergic reactions to dental materials (such as the acrylic mixture used to bond components and denture) are rare but might also occur.

You may experience a change in, or loss of, feeling in your lips, gums, and/or chin after implant placement. This is usually temporary. Some soreness may occur in the gums where the implants are placed, but this discomfort should be minimal and disappear within a few days. An over-the-counter medication (e.g.: aspirin, Tylenol) should control that pain. No risks or complications are expected from the x-rays.

Your lower denture might break after the installing retentive components inside it. If this happens during the study timeline, we will fix/repair it at no cost.

Losing an implant is uncommon but possible. If your implant is lost or does not attach to the bone, it will be replaced. If it happens, you might opt to return to a conventional denture; we will do it for you if

needed. Implants can become loose, causing the gums to become sore or infected. If this occurs, the implant will be removed and, if necessary, an antibiotic can be taken for the infection.

If you have questions/concerns about anything that does not feel/look right, please contact us as soon as possible.

## 6. Benefits

You may or may not benefit personally from taking part in this project. One expected benefit is better stability/tightness in the fit of your lower denture. It is possible that you will be able to chew better and have a more comfortable lower denture fit. All treatment involved in this study (implant, adapting your denture and changing components during the 18-month period) will be done free of charge. You will benefit from having a dentist examine the health of your mouth, including your gums and tongue. This study will provide researchers with new information about the use of new dental treatment methods and materials for the elderly, and the results of this study may help develop better and more affordable treatments for lost teeth.

You must expect, however, that dentures will not completely eliminate all impairments caused by teeth loss and that results will vary between participants.

## 7. Financial Compensation

As compensation for travel expenses to/from the clinic for the 3 data collection appointments (visits 4, 5 and 6 in the Table), you will receive $25 at each visit, for a total of $75 for 3 visits.

## 8. Protection of confidentiality:

During your participation in this project, the research team will gather information about you in some forms required to meet the project's scientific goals.

All data relating to your participation in this study will be kept strictly confidential and will be protected with a code. The key code connecting your name to your research file will be kept by the researcher responsible. Research data will be kept in the office of Dr. de Souza, principal investigator for McGill University. The data will be kept secured for 25 years after the end of the study and will then be destroyed. Similarly, a code will also be assigned to your sample material (such as implant connectors) that will link it to your name. To ensure the confidentiality of this code and to prevent revealing your name or identity,

only the study staff will know this code. The name that corresponds to the code will be kept in a password-protected computer file in the Division of Oral Health and Society, accessible only by Dr. Raphael de Souza, at McGill University, Faculty of Dentistry, Room 534. Only authorized members of the research team will have access to it.

Members of the McGill Institutional Review Board, or persons designated by this Board, may access the study data to verify the ethical conduct of this study.

## 9. Communication of results

Once the study is completed, we plan to inform the study participants and the general public of the results of the study, in order to improve the treatment of teeth loss in the elderly. Thus, a summary of the study results will be shared with you via email if you wish, or conventional mail if you do not use or do not have access to email or internet. The results will also be available to you and to the general public through social media and the web, with focus on dental professionals.

The results of this research will be presented at scientific meetings and published in scientific journals. No study participant names or identifiable information will be disclosed through these methods. Photographs of your mouth and dentures could be presented for scientific purposes, but they will not show your identity.

## 10. Voluntary participation and right of withdrawal

Your participation is voluntary and, by signing this consent form, you are not waiving any of your rights. In addition, by signing the form, you are not releasing the researchers from their legal and professional responsibilities and liability.

You may leave or withdraw from this study at any time, without giving any reason. You simply have to notify the contact person in the research team, and this can be done verbally or in writing. In case of withdrawal, the information that had been collected up until the time of your withdrawal will not be destroyed so as not to jeopardize the integrity of the study. Throughout the study, you will be notified of any new information that might make you reconsider your participation in the study. Your participation, refusal to participate or withdrawal from the study will have no impact on the care that you receive at the dental clinics of the McGill University.

## 11. Discontinuation of the study

The researcher in charge of the project or the Research Ethics Committee may terminate your participation, without your consent, if new findings or information indicates that your participation in the project is not in your interest, if you do not follow the instructions of the research project or if there are administrative reasons to abandon the project.

## 12. Contact information for questions

1- Questions about the study:

For more information about this research or if you want to withdraw from the study, you can contact Dr. Raphael de Souza, the principal investigator at McGill University, by telephone at (514) 398-4777 or by email at: raphael.desouza@mcgill.ca.

You can also contact the research coordinator, Mr. Nicolas Drolet by telephone at (514) 398-7203, ext. 0199, or by email at: nicolas.drolet@mcgill.ca.

2- Questions about your rights and how to make a complaint:

For more information on ethical issues concerning your participation in this project, please contact the Research Ethics Officer at McGill University (Mrs. Ilde Lepore) by email: ilde.lepore@mcgill.ca or by telephone at (514) 398-8302. Any complaints about your participation in this research project may be addressed to the same person. This reference person accepts collect calls, speaks English and French, and takes calls between 9am and 5pm.

**CONSENT FORM:**

The research project described in this consent form has been explained to me. I am aware of the purpose of this study, aware of what I am being asked to do, and the risks and benefits of taking part in the study. All questions I had about this study have been answered, and I can get more information at any time during the study. I am aware that I can withdraw from this study at any time. I consent and agree to take part in this study. I do not give up any of my legal rights by signing this consent form. I will receive a copy of this consent form for my records.

| Name of participant (printed) | Signature of participant<br><br>Date : |
|---|---|

Storing contact information (optional)
I agree that my contact information be securely stored and that I could be contacted to participate in a future research project. ☐ Yes ☐ No

**Commitment and signature of the researcher:**
I, Raphael F. de Souza, declare to have explained the purpose, nature, benefits and risks of the study, and to have answered all the questions of the participant. The research team and I agree to comply with the terms of this consent form. A signed copy of this document has been given to the participant.

Signature ……………………………………………. Date………………………………….....................

Centre universitaire de santé McGill | McGill University Health Centre

X HME MCH X HGM MGH X HRV RVH
X HNM MNH X ITM MCI X CL LC

*FMU-9997*

Centre universitaire de santé McGill | McGill University Health Centre

## INFORMATION AND CONSENT FORM

| | |
|---|---|
| **Research Study Title:** | Single-Implant Overdentures Retained by the Novaloc Attachment System: a Mixed Methods Randomized Cross-Over Trial |
| **Protocol number:** | 2018-3873 |
| **Researcher responsible for the research study:** | Dr. Raphael F de Souza<br>Faculty of Dentistry, McGill University |
| **Co-Investigator(s)/sites:** | Dr. Nicholas Makhoul (McGill University Health Center, Dentistry and Oral and Maxillofacial Surgery)<br>Professor Jocelyne Feine, Dr. Shahrokh Esfandiari, Dr. Christophe Bedos, Dr. Samer Abi Nader, and Dr. Didem Dagdeviren (Faculty of Dentistry, McGill University) |
| **Sponsor:** | ITI (International Team for Implantology), grant 1185-2016 |

## INTRODUCTION

We are inviting you to take part in this research study because you have no natural teeth and currently wear full dentures. Also, you wish to hold your lower denture better on the jaw by using implants. Regular dentures tend to be less stable for the lower jaw; many people have difficulties wearing their lower dentures because of looseness and discomfort. You should be 65 years or older, because most people with full dentures are seniors. Plus, seniors tend to: (1) have smaller lower jawbones that give weak support to a regular denture; (2) prefer a small number of implants (as we will use), just to hold a denture better. Other reasons are: your health, lower jawbone size and oral hygiene allow us to place a dental implant.

However, before you accept to take part in this study and sign this information and consent form, please take the time to read, understand and carefully examine the following information. You may

also want to discuss this study with your family doctor, a family member or a close friend.

This form may contain words that you do not understand. We invite you to speak to the researcher responsible for this study (the “study doctor”) or to other members of the research team, and ask them to explain to you any word or information that is unclear to you before you sign this form.

**BACKGROUND**

For many years, dentists have provided full dentures for people with no teeth. However, dentures only stay in the mouth by holding onto the gums and bone. Many denture wearers can wear an upper denture easily, but have difficulties wearing their lower dentures because of looseness and discomfort. Without teeth, jawbones shrink, causing further loosening of dentures. This makes it hard for people to chew and speak.

Studies have shown that people who wear lower dentures are more satisfied with them when dental implants support them. Dental implants can make a lower denture more stable and improve chewing and comfort. Implants are shaped like dental roots, inserted in the jawbone, and dentures snap onto them. The technical name of this type of denture is “overdenture”.

Elderly patients are most likely to have no teeth and little remaining bone; a lack of a strong bone foundation makes it difficult to securely place implants. In addition, the cost of implant treatment is usually far beyond the means of most elderly people. Therefore, using a minimum number of implants, such as a single implant in the front part of the lower jaw where enough bone is most likely to remain, will also lower the cost. The denture can be attached to the implant using a connector that holds the denture tight. Recent studies show that a single implant can stay in place for the same time as with more than one implant.

We will compare a new type of connector to another often used around the world; both are attached with a single implant in the lower jaw. We will invite you to rate your satisfaction with both connectors and describe your experiences with each connector in individual interviews. We will calculate the costs for each type determine their cost-benefit. Our results will guide dentists and patients when choosing the most appropriate connector for their needs.

**PURPOSE OF THE RESEARCH STUDY**

The purpose of this study is to learn about your satisfaction with a new implant connector type called Novaloc. We will attach it to a single implant in your lower jaw, and compare it to a traditional type called Locator. We will also ask which would be your preferred connector. We plan to ask you why you prefer one of these connectors in an interview.

Secondary purposes are to compare connectors according to: (1) how well your dentures will work from the dentist’s viewpoint; (2) estimated cost of the treatment.

For this research study, we will recruit 26 participants, men and women, aged 65 years or more.

## DESCRIPTION OF THE RESEARCH PROCEDURES

This research study will take place at two sites:
1. Satellite clinic at the Department of Dentistry, Montreal General Hospital (1650 Cedar Ave, 3rd floor, H3G 1A4, Montreal, QC)
2. Faculty of Dentistry, McGill University (2001 McGill College Ave, 1st floor, H3A 1G1, Montreal, QC)

### 1. Duration and number of visits

Your participation in this research project will last 18 months and will include 06 visits. Each visit will last around 90 minutes.

### 2. Study devices

When participating in this research project, you will be assigned to one of the following groups:

Group 1: Novaloc component, followed by the traditional (Locator) component (3 months each)
Group 2: Traditional (Locator) component, followed by Novaloc (3 months each)

The traditional component used in this research study looks very similar to Novaloc, except for the color and few details in shape. We are using a well-known component to compare with the new type (Novaloc) to ensure that the differences you report for them, good or bad, are not only due to chance. Plus, we will be able to see if Novaloc really provides advantages to justify its use (it is more expensive than Locators). In this information and consent form, the use of "study device" refers either to the implant component of interest (Novaloc) or to the traditional component (Locator). After you try both study devices, we will ask you to choose one to keep.

Furthermore, this study is randomized which means that you will be assigned to one of the groups. You may not choose the group to which you will be assigned; this process is done randomly like flipping a coin. One person out of 2 (50%) will receive Novaloc first whereas one person out of 2 (50%) will receive Locator first.

This is a single blind study, which means that the researchers who will make questions will not know which component you will have during this project. In case of emergency, the study doctor will have access to this information.

### 3. Tests and procedures

During your participation in this research study, the study doctor or a member of the research team will conduct the following tests and procedures:

| DESCRIPTION OF STUDY PROCEDURES | |
|---|---|
| **Procedure** | **Description** |
| 1. Screening | - Initial questionnaire, mouth exam *(as the standard of care)*<br>- Consent form *(only for the study)* |
| 2. X-rays | - X-rays to confirm that your jaw can receive implants *(as the standard of care)* |
| 3. Implant insertion | - Minor oral surgery for insertion of implant: we will numb your lower jaw to prevent pain. Then, we will prepare the jawbone and install the implant. We will place some stiches, and grind your denture above the implant *(as the standard of care)* |
| 4. Implant follow-up | - A brief appointment to see if your implant and surrounding gum are fine (a week after insertion) *(as the standard of care)* |
| 5. Prosthetic modification | - Hollowing your denture and installing retentive components *(as the standard of care)*. This will be done at least 8 weeks after implant insertion. |
| 6. Data collection | - Questionnaires and mouth exam *(only for the study)* |
| 7. Changing components | - Grinding the denture base to remove a component and place another *(only for the study)* |
| 8. Interview | - You will discuss your feelings and preferences with the components and implant. This will happen in private and out of the clinic *(only for the study)* |

The schedule of procedures for each visit is listed below:

| SCHEDULE OF STUDY PROCEDURES | | | | | | | |
|---|---|---|---|---|---|---|---|
| **Procedure** | **Visit 1** | **Visit 2** | **Visit 3** | **Visit 4** (Day 0) | **Visit 5** (3 months) | **Visit 6** (6 months) | **Visit 7** (18 months) |
| 1. Screening | X | | | | | | |
| 2. X-rays | X | | | | | | |
| 3. Implant insertion | | X | | | | | |
| 4. Implant follow-up | | | X | | | | |
| 5. Prosthetic modification | | | | X | | | |
| 6. Data collection | | | | X | X | X | X |
| 7. Changing components | | | | | X | X* | |
| 8. Interview | | | | | | X | |
| **Study site** | **Montreal General Hospital** | | | **Faculty of Dentistry, McGill University** | | | |

* We may change the component or not, depending on your preference.

## PARTICIPANT'S RESPONSIBILITIES

- As for standard dental treatment, please communicate if you cannot come to any of the scheduled appointments. Plus, please tell us if something changes in your health, even if that may not seem relevant.
- Dentures and implants need regular care; please brush them at least twice/daily. We recommend you to see a dentist regularly after the end of the study, at least once/year.
- You will be responsible for the costs of dental care after the study ends. Eventual repairs and changes of components will be necessary, as normal for dentures and implants. Fees may vary

in different clinics, but you may expect fees of nearly $100 for repairing a component. Other repairs may be more expensive, such as refitting the denture with acrylic ($394 or more, depending on how it is done).

**BENEFITS ASSOCIATED WITH THE RESEARCH STUDY**

You may or may not personally benefit from your participation in this research project. However, we hope that the study results will contribute to the advancement of scientific knowledge in this field and help us find better treatments for patients.

All treatment involved in this study (implant, adapting your denture and changing components during the 18-month period) will be done free of charge.

**RISKS ASSOCIATED WITH THE RESEARCH STUDY**

Both study devices are licensed for patient use and sold in Canada and United States. This way, risks associated with treatment are the same expected for minor oral surgery and standard dental implants/dentures.

If you have noticed side effects, whatever they may be, during this research study, you must tell the study doctor immediately, regardless of whether you think these effects are related to the implants or components. Even once your participation in the study is over, do not hesitate to contact the study doctor if you experience a side effect that may be linked to the study devices.

The study doctor and members of his or her team will answer any questions that you may have regarding the risks, discomforts and side effect associated with this study. Also, at each visit, the study doctor and members of his or her team will ask you questions about any side effects you may have experienced.

**1. Risks associated with implant insertion**

Prior to implant placement, you will receive an anesthetic injection to reduce sensitivity to pain. An allergic reaction to the injection is rare, although possible. If this occurs, safe, standard procedures will be followed. These could include providing anti-histamines and / or adrenaline.

You may experience a change in, or loss of, feeling in your lips, gums, and/or chin after implant placement. This is usually temporary. Some soreness may occur in the gums where the implants are placed, but this discomfort should be minimal and disappear within a few days. An over-the-counter medication (e.g.: aspirin, Tylenol) should control that pain.

**2. Risks associated with dentures**

You may experience some sore spots under your dentures after the placement of implants or retentive

components. If this happens, the dentures will be adjusted for you as necessary. Allergic reactions to dental materials (such as the acrylic mixture used to bond components and denture) are rare but might also occur.

Your lower denture might break after the installing retentive components inside it. If this happens during the study timeline, we will fix/repair it at no cost.

**3. Other risks**

Losing an implant is uncommon but possible. If your implant is lost or does not attach to the bone, it will be replaced. If it happens, you might opt to return to a conventional denture; we will do it for you if needed. Implants can become loose, causing the gums to become sore or infected. If this occurs, the implant will be removed and, if necessary, an antibiotic can be taken for the infection.

We do not expect risks or complications from the x-rays or other exams. This includes data collection and interviews.

**RISKS ASSOCIATED WITH PREGNANCY**

This study presents no risk of this type due to the minimum age for participation (65 years).

**OTHER POSSIBLE TREATMENTS**

You do not have to take part in this study to receive medical care for your condition. Other options exist such as: (1) new full dentures or repairs (“relinings”); (2) more than one implant in the lower jaw; (3) implants in the upper jaw. We encourage you to discuss with the study doctor all available options.

**VOLUNTARY PARTICIPATION AND THE RIGHT TO WITHDRAW**

Your participation in this research project is voluntary. Therefore, you may refuse to participate. You may also withdraw from the project at any time, without giving any reason, by informing the study doctor or a member of the research team.

Your decision not to participate in the study, or to withdraw from it, will have no impact on the quality of care and services to which you are otherwise entitled, or on your relationship with the study doctor or clinical team.

The study doctor, the Research Ethics Board, the funding agency, or the Sponsor may put an end to your participation without your consent. This may happen if new findings or information indicate that participation is no longer in your interest, if you do not follow study instructions, or if there are administrative reasons to terminate the project.

However, for safety and future result analyses, before you withdraw from the study we ask you to

notify the contact person in the research team, verbally or in writing.

If you withdraw or are withdrawn from the study, the information and biological material already collected for the study will be stored, analyzed and used to ensure the integrity of the study.

Any new findings that could influence your decision to stay in the research project will be shared with you as soon as possible.

**CONFIDENTIALITY**

During your participation in this study, the study doctor and their team will collect and record information about you in a study file. They will only collect information required to meet the scientific goals of the study.

The study file may include information from your medical chart, including your identity, concerning your past and present state of health, your lifestyle, as well as the results of the tests, exams, and procedures that you will undergo during this research project. Your research file could also contain other information, such as your name, sex, date of birth and ethnic origin.

The research forms and x-rays will be sent to Dr. de Souza's office and stored for 25 years for the exclusive objectives of this study and then destroyed. His office is located at McGill University, Faculty of Dentistry, 2001 McGill College Ave, suite 534, H3A1G1, Montreal (QC).

All the information collected during the research project will remain strictly confidential to the extent provided by law. You will only be identified by a code number. The key to the code linking your name to your study file will be kept by the study doctor.

To ensure your safety, a copy of this information and consent form (including the type of implant/components in use and x-ray results) will be placed in your medical chart. As a result, any person or company to whom you give access to your medical chart will have access to this information.

The study doctor will forward your coded data to the sponsor or their representatives.

The Sponsor may share the coded study data with their commercial partners. However, the sponsor and any international partners will respect the confidentiality rules in effect in Quebec and Canada, regardless of the country to which your data may be transferred.

The study data will be stored for 25 years by the principal investigator (Dr de Souza).

The data may be published or shared during scientific meetings; however it will not be possible to identify you.

For monitoring, control, safety, security, and marketing of a new study drug, your study file as well as your medical charts may be examined by a person mandated by Canadian or international regulatory

authorities, such as Health Canada, as well as by representatives of the study sponsor, the institution, or the Research Ethics Board. All these individuals and organizations adhere to policies on confidentiality.

You have the right to consult your study file in order to verify the information gathered, and to have it corrected if necessary.

However, in order to protect the scientific integrity of the research project, accessing certain information before the project is ended may require that you be withdrawn from the study.

**INCIDENTAL FINDINGS**

Material incidental findings are findings made in the course of the study that may have significant impacts on your current or future wellbeing or that of your family members. A material incidental finding concerning you in the course of this research will be communicated to you and to a health professional of your choice.

We will exam you according to standard practices in dentistry during “visit 1”. This may reveal certain diseases that are outside our study goals but have importance for your well-being. Examples are: (1) infection of your mouth; (2) tumours or cysts only visible by x-ray. We will tell you about such a finding and refer you to adequate treatment when needed. This will happen regardless of your inclusion or not in this study. If we have any incidental finding during any visit, we will do the same.

**MARKETING POSSIBILITIES**

The research results, including those following your participation in this study, could lead to the creation of commercial products. However, you will not receive any financial benefits.

**FUNDING OF THE RESEARCH PROJECT**

The study doctor and the institution have received funding from the sponsor for the completion of the research project.

**COMPENSATION**

You will receive an amount of 25$ per study visits 5, 6 and 7, for a total of 03 visits, for a total amount of 75$ for costs and inconveniences incurred during this research study. If you withdraw from the study, or are withdrawn before it is completed, you will receive compensation proportional to the number of visits you have completed. Implant insertion and placement of components will be offered to you for free for the duration of this research study.

**SHOULD YOU SUFFER ANY HARM**

Should you suffer harm of any kind following administration of the study drug, or following any other

procedure related to the research study, you will receive the appropriate care and services required by your state of health.

By agreeing to participate in this research project, you are not waiving any of your legal rights nor discharging the study doctor, the sponsor or the institution, of their civil and professional responsibilities.

**CLINICAL TRIAL REGISTRATION**

A description of this clinical trial is available on http://www.ClinicalTrials.gov (project NCT03126942). This Website will not include information that can identify you. At most, the Website will include a summary of the results. You can search this Website at any moment, or access https://clinicaltrials.gov/ct2/show/NCT03126942 directly.

**CONTACT INFORMATION**

If you have questions or if you have a problem you think may be related to your participation in this research study, or if you would like to withdraw, you may communicate with the study doctor or with someone on the research team at the following number: Mr. Nicolas Drolet, telephone: (514) 398-7203, ext. 0199; email: nicolas.drolet@mcgill.ca; or Dr. Raphael de Souza (principal investigator) at McGill University, telephone: (514) 398-4777; email: raphael.desouza@mcgill.ca.

For any question concerning your rights as a research participant taking part in this study, or if you have comments, or wish to file a complaint, you may communicate with:

(1) The Patient Ombudsman of the Montreal General Hospital at the following phone number: (514) 934-1934, ext. 44285.

(2) the Research Ethics Officer at McGill University (Mrs. Ilde Lepore) by email: ilde.lepore@mcgill.ca or by telephone at (514) 398-8302.

**OVERVIEW OF ETHICAL ASPECTS OF THE RESEARCH**

The McGill University Health Centre Research Ethics Board reviewed this study and is responsible for monitoring it at all participating institutions in the health and social services network in Quebec.

**Research Study Title:** Single-Implant Overdentures Retained by the Novaloc Attachment System: a Mixed Methods Randomized Cross-Over Trial

**SIGNATURES**

***Signature of the participant***

I have reviewed the information and consent form. Both the research study and the information and consent form were explained to me. My questions were answered, and I was given sufficient time to make a decision. After reflection, I consent to participate in this research study in accordance with the conditions stated above.

I authorize the research study team to have access to my medical record for the purposes of this study.

- I authorize the doctor in charge of this research study to communicate with me directly to ask if I am interested in participating in other research:

  Yes ☐
  No ☐

- I authorize the study doctor to inform my treating physician that I am taking part in this study:

  Yes ☐ Name and contact information of treating physician: ______________________________
  No ☐
  I do not have a treating physician/I am no longer being followed by my treating physician ☐

I understand that the study doctor will send my treating physician health information if it will be useful for my care.

_______________________________________________________________

Name of participant Signature Date

***Signature of the person obtaining consent***

I have explained the research study and the terms of this information and consent form to the research participant, and I answered all his/her questions.

_______________________________________________________________

Name of the person obtaining consent Signature Date

***Commitment of the principal investigator***

I certify that this information and consent form were explained to the research participant, and that

the questions the participant had were answered.

I undertake, together with the research team, to respect what was agreed upon in the information and consent form, and to give a signed and dated copy of this form to the research participant.

______________________________________________________________________

Name of the principal investigator Signature Date

Faculté de médecine dentaire

# FORMULAIRE D’INFORMATION ET DE CONSENTEMENT

## Prothèses dentaires complètes à recouvrement retenues par un implant unique et attachements Novaloc : un essai clinique croisé à méthodes mixtes.

**Chercheur principal :**
D[r] Raphael F de Souza, D.D.S., M.Sc., Ph. D.
Faculté de médecine dentaire, Université McGill
2001, avenue McGill College, bureau 534, Montréal (Québec) H3A 1G1
Tél :(514) 398-4777; Courriel : raphael.desouza@mcgill.ca

**Membres de l'équipe de recherche :** D[r] Jocelyne Feine, D[r] Shahrokh Esfandiari, D[r] Nicholas Makhoul, D[r] Christophe Bedos, D[r] Samer Abi Nader, et D[r] Didem Dagdeviren
Faculté de médecine dentaire, Université McGill

## 1. Objectifs de ce formulaire de consentement

Nous vous invitons à participer à ce projet de recherche dont le but sera de déterminer la réussite d’une méthode de traitement avec des implants dentaires, et comparer deux connecteurs utilisés sur des implants pour maintenir en place les prothèses dentaires complètes. Les informations obtenues seront utiles pour mieux soigner les personnes âgées qui n'ont plus de dents. Ce formulaire sert à :

a. Vous informer, de façon aussi exhaustive que possible, de la nature et de l’objectif de l’étude ainsi que des risques inhérents à ladite étude;
b. Vous fournir tous les renseignements dont vous avez besoin pour décider si vous souhaitez participer ou non à l’étude, selon vos objectifs personnels;
c. Vous expliquez votre condition de santé buccale et son traitement.

Veuillez lire ce formulaire de consentement attentivement et poser toute question que vous jugez nécessaire avant de décider si vous souhaitez ou non prendre part à cette étude. Les chercheurs sont ici pour vous aider à comprendre le processus à fond et n’hésitez donc pas à leur demander quoi que ce soit relativement à l’étude. Veuillez prendre tout le temps dont vous avez besoin et n’hésitez pas à discuter de cette étude avec votre famille ou vos amis avant de prendre votre décision. Votre participation à l’étude est entièrement volontaire et, si vous décidez de ne pas y prendre part, vous ne ferez l’objet d’aucune

pénalité et vous ne perdrez aucun des avantages auxquels vous avez droit.

Vous ne devriez PAS prendre part à cette étude si vous préférez porter des prothèses traditionnelles ou êtes tout à fait satisfait de vos prothèses actuelles.

## 2. Introduction

Pendant de nombreuses années, les dentistes ont fourni les prothèses dentaires amovibles comme traitement pour les personnes qui n'ont plus de dents. Cependant, la stabilité de ces prothèses dépend uniquement de son contact avec les gencives et les mâchoires. Les personnes qui portent des prothèses complètes sont normalement capables de porter aisément une prothèse supérieure, mais plusieurs éprouvent des difficultés à porter une prothèse inférieure parce qu'elle bouge et devient une source d'inconfort. La taille des mâchoires diminue avec la perte des dents. Cela diminue ainsi la stabilité des prothèses dentaires et gêne la mastication et l'élocution.

Les études ont révélé que les personnes qui portent des prothèses inférieures en sont beaucoup plus satisfaites lorsque des implants dentaires les soutiennent. L'implant dentaire est comme une racine dentaire qui est ancrée dans l'os des mâchoires et sert à attacher une prothèse dentaire.

Les personnes âgées sont les plus susceptibles à n'avoir aucune dent et ont souvent des petites mâchoires, ce qui rend la pose d'implants plus difficile. De plus, le coût des implants dentaires est habituellement trop élevé pour plusieurs aînés. Donc, un nombre minimal d'implants, c'est-à-dire un seul implant dans la région antérieure de la mâchoire inférieure (où la taille de l'os est souvent suffisante), peut réduire les coûts de traitement. La prothèse va s'attacher à l'implant au milieu d'un connecteur qui va fournir un serrage additionnel.

Plusieurs dentistes regardent les deux implants comme un nombre minimal pour attacher une prothèse inférieure. On croyait qu'un seul implant ne pourrait pas durer en bouche pendant longtemps ou retenir une prothèse complète assez fortement. Cependant, des études récentes montrent qu'un seul implant peut durer le même temps observé pour les nombres différents. Chez les patients édentes, la pose d'un ou deux implants conduits à la même satisfaction de leurs prothèses inférieures.

Nous allons comparer deux types de connecteur. Le premier est déjà utilisé couramment dans le monde entier (Locator). Le deuxième est nouveau et s'appelle Novaloc. Ces deux connecteurs seront posés, chacun leur tour, dans la mâchoire inférieure. Les participants évalueront leur satisfaction avec les deux

connecteurs et décriront leurs expériences avec chacun pendant une entrevue. Les coûts de traitement avec chaque connecteur seront estimés pour définir le coût-bénéfice de chacun. Les résultats vont aider les dentistes et les patients à choisir le meilleur connecteur selon leurs besoins.

## 3. Objectifs

Le principal objectif de cette étude est:

- Connaître votre niveau de satisfaction face à votre prothèse inférieure avec un nouveau connecteur (appelé Novaloc) ancré sur un seul implant, comparé à un connecteur traditionnel (Locator). Nous voudrions également savoir lequel sera votre connecteur préféré et les raisons pour lesquelles vous l'avez préféré.

Comme objectifs secondaires, nous allons comparer les deux connecteurs selon:

- La qualité de vie et comment vous percevez le fonctionnement des prothèses et connecteurs;
- La qualité de vos prothèses dentaires évalué par le dentiste. Cela va inclure les besoins en réparations et la survie des implants ainsi que l'efficacité des attachements prothétiques et leurs composantes;
- Coût estimé des traitements fournis.

## 4. Déroulement du projet

Cette étude inclura environ 26 participants. Vous pouvez être admissible à participer si vous:

- Êtes âgés de 65 ans ou plus et n'avez aucune dent naturelle en bouche depuis au moins 6 mois;
- Portez des prothèses dentaires complètes en bonne état, et voulez avoir la prothèse inférieure ancrée sur un implant dentaire;
- Possédez une mâchoire inférieure suffisamment grande. L'os de la région antérieure doit présenter des dimensions suffisantes pour recevoir l'implant;
- Êtes relativement en bonne santé et en mesure de nettoyer vos prothèses dentaires. Veuillez nous dire si vous portez quelque maladie ou utilisez des médicaments (comme les m médicaments destinés à fluidifier le sang ou pour renforcer l'os).

Si vous vous portez volontaire pour participer à cette étude, nous ferons quelques examens et remplirons des formulaires. Ces procédures sont similaires à celles qui sont faites de façon routinière avant l'insertion d'implants dentaires. Cela inclura des questions sur votre santé générale et buccale, l'état de santé de votre bouche et des radiographies, c'est-à-dire une tomographie assistée par ordinateur. Les résultats de ces examens seront comparés à une liste de critères d'inclusion requis pour la participation. Votre admission sera confirmée si vos résultats répondent aux critères.

Vous saurez si vous pouvez participer dès la première visite. Si vous confirmez votre intérêt, nous aurons besoin de votre signature à ce formulaire de consentement. Veuillez emporter ce formulaire avec vous afin d'en parler à votre famille, dentiste, denturologiste ou autres personnes, si vous le souhaitez. Après avoir signé ce formulaire de consentement, nous allons collecter quelques données tel que mentionné dans la section « Objectifs », et vous donner un rendez-vous pour insérer l'implant.

Vous allez recevoir l'implant à la deuxième visite (diamètre : 3.3 mm; longueur minimale : 10 mm). La pose de l'implant se fera sous anesthésie locale. L'os de la mâchoire inférieure sera préparé et l'implant sera posé par la suite. Nous ferons des points de suture et la prothèse dentaire sera évidée et placée au-dessus de l'implant et les points. Nous attendrons la guérison de l'os durant huit semaines ou plus. Environ une semaine après la pose de l'implant, nous vous demanderons de venir pour au moins une visite, dont le but sera vérifier la guérison de votre bouche.

Après la période de guérison de huit semaines, nous poserons l'attachement qui ancrera votre prothèse dentaire inférieure. Nous visserons une partie métallique cylindrique dans votre implant, et placerons une autre partie dans la prothèse – c'est-à-dire, une partie qui contient une pièce de plastique. La pièce de plastique va s'attacher autour de la partie métallique sur l'implant pour fournir une meilleure stabilité à votre prothèse. Chaque type de connecteur (Novaloc ou Locator) est composé de différents matériaux et formes. Ils sont composés de différentes pièces de plastiques ainsi que des parties métalliques distinctes sur l'implant.

**Vous recevrez chacun des deux connecteurs aléatoirement / par chance** (nous compterons ce temps comme le début de l'étude). La base de votre prothèse dentaire inférieure sera évidée pour créer de l'espace suffisant pour l'attachement de rétention. Par à la suite, nous utiliserons une résine acrylique pour ancrer l'attachement dans la prothèse.

**Trois mois après** avoir reçu le premier connecteur, nous collecterons les données mentionnées dans les « Objectifs » (questionnaires remplis par vous et le dentiste), sauf l'entrevue sur vos préférences. Vous recevrez l'autre type de connecteur (si vous avez commencé avec Locator, vous recevrez Novaloc à cette visite, et vice versa). Pour cela, nous enlèverons le premier attachement à l'intérieur de la prothèse et polir doucement l'acrylique autour.

**Après trois autres mois** (temps total : 6 mois), nous collecterons des données exactement de la même façon que la dernière visite. Vous pouvez garder le même connecteur que vous avez ou le changer pour l'autre selon votre préférence. Par la suite, un autre chercheur fera une entrevue avec vous en dehors de la clinique (temps estimé : 1 heure) et vous demandera vos préférences et expériences avec les connecteurs et l'ensemble du traitement reçu.

**Un an après** (temps total : 18 mois), nous collecterons des informations dans la clinique en utilisant les mêmes questionnaires que la visite de trois mois.

Nous prendrons aussi des photos de votre bouche et de vos prothèses dentaires et collecterons les parties du matériel usagés, qui iront à servir comme matériaux d'étude.

La table en-dessous décrit les étapes de cette étude. Des rendez-vous mineurs (par exemple, pour enlever les points de suture, prendre des radiographies ou quelques ajustements de votre prothèse dentaire) seront aussi nécessaires.

| Visite | Étapes de l'étude | Traitements/obtentions de données | Durée (heure) |
|---|---|---|---|
| 1 | Triage | Questionnaire, examen buccal, consentement éclairé | 1,5 |
| 2 | Pose de l'implant | Chirurgie buccale mineure pour insérer l'implant, l'ajustment de votre prothèse au-dessus de l'implant | 1,5 |
| 3 | Modification de la prothèse dentaire | Questionnaire, examen buccal, la prothèse sera évidée et recevra le component d'ancrage | 1,5 |
| 4 | Première collecte de données (3 mois) | Questionnaire, examen buccal, remplacement du component d'ancrage | 1,5 |
| 5 | Deuxième collecte de données (6 mois) | Questionnaire, examen buccal, (remplacement de l'attachement d'ancrage, selon votre désir), entrevue | 2,5 |
| 6 | Troisième collecte de données (18 mois) | Questionnaire, examen buccal | 1 |

Si vous vous retirez de cette étude, même si c'est à la fin, nous vous réfèrerons à un dentiste de votre choix pour des contrôles périodiques et soins dentaires réguliers ou d'urgence. Si vous faites des changements

non planifiés au traitement tels que des prothèses dentaires neuves, remplacement des connecteurs ou la pose d'autres implants (veuillez nous en informer) ceci conduira probablement à votre retrait de l'étude.

## 5. Risques et inconvénients associés

Les risques associés au traitement sont les mêmes que ceux des chirurgies mineurs et prothèse dentaires amovibles déjà connus.

Avant la mise en place des implants, on vous injectera un anesthésiant local. Il est possible quoiqu'improbable que vous ayez une réaction allergique à ce produit. Si cela se produit, on suivra des procédures standard pouvant comprendre des antihistaminiques ou de l'adrénaline.

Vous pourrez ressentir de la douleur à certains endroits de votre prothèse après la mise en place de l'implant. Si cela se produit, vos prothèses dentaires seront ajustées selon le besoin. Des allergies au matériaux dentaires (par exemple, le mélange acrylique utilisé pour fixer l'attachement de rétention à la prothèse dentaire) sont rares mais pas impossible.

Vous pourriez éprouver un changement ou une perte de sensation dans vos lèvres, vos gencives ou votre menton après la mise en place des implants. Ce phénomène est généralement de nature temporaire. Une infection peut aussi se produire, auquel cas il faudra la traiter à l'aide d'antibiotiques au besoin. Vous pourrez ressentir une certaine douleur aux gencives à l'emplacement des implants, mais cet inconfort devrait être minime et disparaître après quelques jours. Vous devriez être en mesure de calmer la douleur grâce à un analgésique sans ordonnance (par exemple, aspirine, Tylenol). Les examens radiographiques n'impliquent aucun risque ni complication.

Votre prothèse dentaire inférieure peut casser après l'insertion des connecteurs. Si cela se passe pendant l'étude, nous réparerons votre prothèse sans frais.

La perte d'implant est peu fréquente mais possible. Si votre implant est perdu ou ne s'ancre pas à l'os, il sera remplacé. Vous avez encore l'option de continuer à porter une prothèse dentaire conventionnelle sans implant. Vous pouvez retourner à cette prothèse si vous le souhaitez. Les implants dentaires peuvent se détacher graduellement de l'os et provoquer l'endolorissement ou de l'infection. Si cela se produit, l'implant devra être enlevé et nous devrons possiblement vous prescrire un antibiotique pour l'infection.

Si vous avez des questions ou préoccupations concernant un problème qui ne semble pas normal, veuillez nous contacter aussitôt que possible.

## 6. Avantages

Il est possible que vous puissiez profiter personnellement de votre participation à cette étude. Un avantage possible est d'améliorer la stabilité de votre prothèse inférieure. C'est possible que vous soyez en mesure de mieux mâcher et que votre prothèse dentaire inférieure soit plus confortable. Tous les traitements faits pour cette étude (implant, ajustement de votre prothèse et changement des attachements durant 18 mois) n'entraîneront pas de frais. Vous aurez aussi un dentiste qui examinera votre santé buccale, y compris les gencives, la langue et les autres régions. Les renseignements recueillis durant cette étude pourront aider les chercheurs à améliorer la qualité future des soins prodigués aux personnes portant des prothèses dentaires.

Cependant, il faut savoir que les prothèses dentaires ne peuvent pas complètement éliminer les déficiences causées par la perte des dents. Il est probable que les résultats vont varier d'un participant à l'autre.

## 7. Indemnisation financière

Comme indemnisation de vos frais de déplacement pour les 3 visites d'obtentions de données (4e, 5e et 6e visites sur la Table), vous recevrez un montant de 15 $ à chaque visite, soit un total de 45 $ pour les 3 visites.

## 8. Protection de la confidentialité

Au cours de votre participation à ce projet, le chercheur et son équipe recueilleront dans un dossier de recherche des renseignements qui vous concerne. Ces données seront nécessaires à la réalisation des objectifs de l'étude.

Toutes les données relatives à votre participation à cette étude seront strictement confidentielles et se verront attribuer un code. La clé du code associant votre nom à votre dossier de recherche sera conservée par le chercheur responsable. Les données de recherche seront conservées au bureau du Dr de Souza, chercheur principal de l'Université McGill, (2001, avenue McGill Collège, bureau 534). Ces données seront gardées pendant 25 ans suivant la fin de l'étude et seront détruites par la suite.

Un code sera également attribué à votre échantillon (comme les connecteurs sur votre implant et matériaux usés) et associera ce dernier à votre nom. Afin d'assurer la confidentialité de ce code et pour empêcher la divulgation de votre nom ou de votre identité, seul le personnel de l'étude prendra connaissance de ce code. Le nom correspondant au code sera conservé dans un fichier informatique protégé par un mot de

passe, dans la Division de Santé Buccodentaire et Société, à l'Université McGill (Faculté de médecine dentaire, bureau 500). Seuls les membres de l'équipe de recherche autorisés y auront accès.
Seuls les membres du comité d'éthique de la recherche de l'Université McGill, ou les personnes désignées de ce comité pourront accéder aux données obtenues pour vérifier la conduite éthique de l'étude.

## 9. Diffusion des résultats

Nous avons l'intention, une fois l'étude terminée, d'informer les participants de l'étude et le grand public des résultats de l'étude, afin d'améliorer les soins dentaires pour les personnes qui n'ont plus de dents. Ainsi, un résumé des résultats de l'étude vous sera envoyé par courriel ou par la poste traditionnelle si vous n'utilisez pas Internet ou n'avez pas d'accès Internet. Autrement, les résultats seront disponibles pour le grand public à travers les médias sociaux et le Web. Les résultats de cette recherche seront également présentés lors de réunions scientifiques et ils seront publiés dans des revues spécialisés.
Aucune information permettant de vous identifier ne sera divulguée, peu importe le moyen de diffusion des résultats. Les photographies de votre bouche et prothèses pourraient être montrées à des fins scientifiques, mais elles ne permettront pas de vous identifier.

## 10. Participation volontaire et droit de retrait

Votre participation est volontaire et en signant le présent formulaire de consentement, vous ne renoncez à aucun de vos droits prévus par la loi. De plus, vous ne libérez pas les chercheurs de leurs responsabilités juridiques et professionnelles.
Vous pouvez vous retirer de cette étude à tout moment, sans avoir à donner de raison. Il vous suffit d'aviser la personne-ressource de l'équipe de recherche, et ce, par simple avis verbal. En cas de retrait, les renseignements recueillis au moment de votre retrait ne seront pas détruits afin de ne pas mettre en péril l'intégrité de l'étude.
Tout au long de l'étude, vous serez avisé de toute nouvelle information susceptible de vous faire remettre en question votre participation à l'étude.
Votre participation, le refus de participer ou le retrait en cours d'étude n'aura pas d'incidence sur les soins reçus aux cliniques de médecine dentaire de l'Université McGill.

## 11. Interruption de l'essai clinique

Le chercheur responsable du projet de recherche et le comité d'éthique de la recherche peuvent mettre fin à votre participation, sans votre consentement, si de nouvelles découvertes ou informations indiquent que votre participation au projet n'est plus dans votre intérêt, si vous ne respectez pas les consignes du projet de recherche ou s'il existe des raisons administratives d'abandonner le projet.

## 12. Personnes-ressources

1- Questions sur le projet de recherche :

Pour plus d'information concernant cette recherche ou si vous voulez vous retirer de l'étude, vous pouvez communiquer avec le chercheur principal de cette étude, Dr. Raphael de Souza (Université McGill), par téléphone au (514) 398-4777 ou par courriel à : raphael.desouza@mcgill.ca.
Vous pouvez aussi contacter le coordonnateur de recherche, M. Nicolas Drolet au téléphone au (514) 398-7203, ext 0199, ou par courriel à nicolas.drolet@mcgill.ca.

2- Plaintes ou questions sur vos droits à titre de participant :

Pour toute information d'ordre éthique concernant les conditions dans lesquelles se déroule votre participation à ce projet, vous pouvez communiquer avec l'Agente en éthique de l'Université McGill (Mme Ilde Lepore) à : ilde.lepore@mcgill.ca ou au numéro de téléphone (514) 398-8302. Toute plainte relative à votre participation à cette recherche peut être adressée aux mêmes cordonnées. Cette agente acceptent les appels à frais virés, s'exprime en français et en anglais, et acceptent les appels entre 9 h et 17 h.

**FORMULAIRE DE CONSENTEMENT**

Le projet de recherche décrit dans le présent formulaire d'information et de consentement m'a été expliqué. Je comprends le but de cette étude et ce qui me sera demandé, ainsi que les risques et avantages de ma participation. On a répondu aux questions que j'ai posées, et je peux obtenir de plus amples renseignements à tout moment pendant l'étude. Je sais que je peux me retirer en tout temps sans préjudice. Je consens librement à prendre part à cette étude. Le fait que je signe ce formulaire n'entraîne aucune renonciation à mes droits reconnus par la loi. Je recevrai un exemplaire de ce document.

| | |
|---|---|
| Prénom et nom du participant<br>(en caractères d'imprimerie) | Signature du participant<br><br>Date : |

Conservation des données personnelles (optionnel)

Je consens à ce que l'on conserve mes coordonnées et que l'on me recontacte afin de me proposer de participer à un autre projet de recherche. ☐ Oui ☐ Non

**Engagement et signature du chercheur :**

Je, Raphael F. de Souza, déclare avoir expliqué le but, la nature, les avantages, les risques et les inconvénients de l'étude et que j'ai répondu à toutes les questions du participant. Je m'engage avec l'équipe de recherche à respecter ce qui a été convenu au formulaire d'information et de consentement et à en remettre un exemplaire signé au participant.

Signature ……………………………………………. Date………………………………

Centre universitaire de santé McGill | McGill University Health Centre

# FORMULAIRE D'INFORMATION ET DE CONSENTEMENT

| | |
|---|---|
| **Titre du projet de recherche :** | Prothèses dentaires complètes à recouvrement retenues par un implant unique et attachements Novaloc : un essai clinique croisé à méthodes mixtes |
| **Numéro de protocole :** | 2018-3873 |
| **Chercheur responsable du projet de recherche :** | Dr Raphael F de Souza<br>Faculté de médecine dentaire, Université McGill |
| **Co-chercheur(s)/sites :** | Dr Nicholas Makhoul (Centre universitaire de santé McGill, Dentisterie et chirurgie orale et maxillo-faciale)<br>Dr Jocelyne Feine, Dr Shahrokh Esfandiari, Dr Christophe Bedos, Dr Samer Abi Nader, et Dr Didem Dagdeviren (Faculté de médecine dentaire, Université McGill) |
| **Commanditaire :** | ITI (International Team for Implantology), subvention 1185-2016 |

## INTRODUCTION

Nous vous invitons à participer à ce projet de recherche parce que vous n'avez aucune dent naturelle et portez des prothèses dentaires complètes. De plus, vous voulez avoir la prothèse inférieure ancrée sur un implant dentaire pour y fournir une meilleure stabilité. Les prothèses traditionnelles sont souvent moins stables sur la mâchoire inférieure ; plusieurs personnes éprouvent des difficultés à porter une prothèse inférieure parce qu'elle bouge et devient une source d'inconfort. Vous devez être âgés de 65 ans ou plus, parce que la plupart des personnes qui portent des prothèses complètes sont âgées. Encore, les personnes âgées ont tendance à : (1) posséder des petites mâchoires qui diminuent la stabilité des prothèses dentaires ; (2) préférer un nombre minimal d'implants (comme nous utiliserons), pour fournir un serrage additionnel à une prothèse. Les autres raisons sont : votre santé (qui permet une chirurgie buccale mineure), la taille de votre mâchoire inférieure et être en mesure de nettoyer vos prothèses dentaires.

Avant d'accepter de participer à ce projet et de signer ce formulaire d'information et de consentement, veuillez prendre le temps de lire, de comprendre et de considérer attentivement les

renseignements qui suivent.

Ce formulaire peut contenir des mots que vous ne comprenez pas. Nous vous invitons à poser toutes les questions que vous jugerez utiles au chercheur responsable de ce projet de recherche (le « dentiste responsable ») ou à un membre de son équipe de recherche et à leur demander de vous expliquer tout mot ou renseignement qui n'est pas clair.

## RENSEIGNEMENTS GÉNÉRAUX

Pendant de nombreuses années, les dentistes ont fourni les prothèses dentaires amovibles comme traitement pour les personnes qui n'ont plus de dents. Cependant, la stabilité de ces prothèses dépend uniquement de son contact avec les gencives et les mâchoires. Les personnes qui portent des prothèses complètes sont normalement capables de porter aisément une prothèse supérieure, mais plusieurs éprouvent des difficultés à porter une prothèse inférieure parce qu'elle bouge et devient une source d'inconfort. La taille des mâchoires diminue avec la perte des dents. Cela diminue ainsi la stabilité des prothèses dentaires et gêne la mastication et l'élocution.

Les études ont révélé que les personnes qui portent des prothèses inférieures en sont beaucoup plus satisfaites lorsque des implants dentaires les soutiennent. L'implant dentaire est comme une racine dentaire qui est ancrée dans l'os des mâchoires et sert à attacher une prothèse dentaire.

Les personnes âgées sont les plus susceptibles à n'avoir aucune dent et ont souvent des petites mâchoires, ce qui rend la pose d'implants plus difficile. De plus, le coût des implants dentaires est habituellement trop élevé pour plusieurs aînés. Donc, un nombre minimal d'implants, c'est-à-dire un seul implant dans la région antérieure de la mâchoire inférieure (où la taille de l'os est souvent suffisante), peut réduire les coûts de traitement. La prothèse va s'attacher à l'implant au milieu d'un connecteur qui va fournir un serrage additionnel.

Nous allons comparer deux types de connecteur. Le premier est déjà utilisé couramment dans le monde entier (Locator). Le deuxième est nouveau et s'appelle Novaloc. Ces deux connecteurs seront posés, chacun leur tour, dans la mâchoire inférieure. Les participants évalueront leur satisfaction avec les deux connecteurs et décriront leurs expériences avec chacun pendant une entrevue. Les coûts de traitement avec chaque connecteur seront estimés pour définir le coût-bénéfice de chacun. Les résultats vont aider les dentistes et les patients à choisir le meilleur connecteur selon leurs besoins.

## OBJECTIFS DU PROJET DE RECHERCHE

Le but de ce projet est de connaître votre niveau de satisfaction face à votre prothèse inférieure avec un nouveau connecteur (appelé Novaloc) ancré sur un seul implant, comparé à un connecteur traditionnel (Locator). Nous voudrions également savoir lequel sera votre connecteur préféré et les raisons pour lesquelles vous l'avez préféré pendant une entrevue.

Comme objectifs secondaires, nous allons comparer les deux connecteurs selon : (1) la qualité de vos prothèses dentaires évalué par le dentiste; (2) coût estimé des traitements fournis.

Pour la réalisation de ce projet de recherche, nous comptons recruter 26 participants, hommes et femmes, âgés de 65 ans ou plus.

**DEROULEMENT DU PROJET DE RECHERCHE**

Ce projet de recherche se déroulera à deux sites :
1. Clinique satellite au département de la Dentisterie et de la Chirurgie orale et maxillo-faciale, Hôpital General de Montréal (1650, avenue Cedar, 3ème étage, H3G 1A4, Montréal, QC)
2. Faculté de Médecine Dentaire, Université McGill (2001, avenue McGill Collège Ave, 1er étage, H3A 1G1, Montréal, QC)

**1. Durée et nombre de visites**

Votre participation à ce projet de recherche durera 18 mois et comprendra 06 visites. Chaque visite sera d'une durée du 90 minutes.

**2. Dispositifs à l'étude**

En participant à ce projet de recherche, vous serez assigné à l'un des groupes suivants :

Groupe 1 : connecteur Novaloc, suivi par le connecteur traditionnel (Locator) (3 mois chacun).
Groupe 2 : connecteur traditionnel (Locator), suivi par le connecteur Novaloc (3 mois chacun).

Le connecteur traditionnel utilisé dans ce projet de recherche ressemble beaucoup au Novaloc, sauf pour sa couleur et des détails dans son contour. Nous comparerons un connecteur bien connu au nouveau type (Novaloc) pour s'assurer que les différences que vous nous signalez, bons ou mauvais, ne sont pas dus uniquement au hasard. Nous allons aussi vérifier si Novaloc possède des avantages qui justifient son usage (il coûte plus cher que les Locators). Soulignons que tout au long de ce formulaire d'information et de consentement, l'expression « dispositif à l'étude » réfère soit au connecteur à l'étude (Novaloc) ou soit au traditionnel (Locator). Après avoir essayé les deux dispositifs à l'étude, vous garderez le connecteur de votre préférence.

Ce projet de recherche est également randomisé, ce qui signifie que vous serez assigné à l'un ou l'autre des groupes. Votre assignation à l'un ou à l'autre de ces groupes relève du hasard, vous ne pourrez donc pas choisir votre groupe. Une personne sur deux (50 %) recevra Novaloc au début et une personne sur deux (50 %) recevra Locator initialement.

Ce projet de recherche est mené à simple insu, ce qui signifie que les membres de l'équipe de recherche qui poseront les questions ne sauront pas quel dispositif vous recevrez durant le projet. Cependant, en cas d'urgence, le dentiste responsable de ce projet de recherche aura ces informations.

**3. Examens et procédures**

Durant votre participation à ce projet, le dentiste responsable de ce projet de recherche ou un membre de son équipe de recherche effectueront les examens et analyses suivants :

| DESCRIPTION DES PROCÉDURES DE L'ÉTUDE | |
|---|---|
| **Procédure** | **Description** |
| 1. Triage | - Questionnaire initial, examen buccal *(soins standards)*<br>- Consentement éclairé *(effectué pour la recherche)* |
| 2. Rayon-X | - Rayons-X pour confirmer si votre mâchoire présente des dimensions suffisantes pour recevoir un implant *(soins standards)* |
| 3. Pose de l'implant | - Chirurgie buccale mineure pour poser l'implant: la pose se fera sous anesthésie locale. Par la suite, l'os de la mâchoire inférieure sera préparé et l'implant sera posé. Nous ferons des points de suture et la prothèse dentaire sera évidée et placée au-dessus de l'implant et les points *(soins standards)* |
| 4. Suivi de l'implant | - Une bref visite pour vérifier la guérison de votre bouche, environ une semaine après la pose de l'implant *(soins standards)* |
| 5. Pose de connecteur | - La base de la prothèse inférieure sera évidée pour créer de l'espace suffisant pour le connecteur. Par la suite, nous utiliserons une résine acrylique pour ancrer l'attachement dans la prothèse *(soins standards)*. Cela se passera après la période de guérison d'au moins huit semaines. |
| 6. Collecte des données | - Questionnaires et examen buccal *(effectué pour la recherche)* |
| 7. Echange de connecteur | - Nous enlèverons le premier attachement à l'intérieur de la prothèse et poserons un autre type *(effectué pour la recherche)* |
| 8. Entrevue | - Un chercheur vous demandera vos préférences et expériences avec les connecteurs et l'implant. Cela se passera en privé et dehors de la clinique *(effectué pour la recherche)* |

Le calendrier des procédures de chaque visite est listé ci-dessous:

| CALENDRIER DES PROCÉDURES DE L'ÉTUDE | | | | | | | |
|---|---|---|---|---|---|---|---|
| **Procédure** | **Visite 1** | **Visite 2** | **Visite 3** | **Visite 4 (Jour 0)** | **Visite 5 (3 mois)** | **Visite 6 (6 mois)** | **Visite 7 (18 mois)** |
| 1. Triage | X | | | | | | |
| 2. Rayon-X | X | | | | | | |
| 3. Pose de l'implant | | X | | | | | |
| 4. Suivi de l'implant | | | X | | | | |
| 5. Modification de la prothèse dentaire | | | | X | | | |
| 6. Collecte de données | | | | X | X | X | X |
| 7. Echange de connecteur | | | | | X | X* | |
| 8. Entrevue | | | | | | X | |
| **Site d'étude** | **Hôpital General de Montréal** | | | **Faculté de Médecine Dentaire, Université McGill** | | | |

* Vous pouvez garder le même connecteur que vous avez ou le changer pour l'autre selon votre préférence.

## VOTRE COLLABORATION AU PROJET DE RECHERCHE

- Pour les soins dentaires standards, veuillez nous dire si vous ne pouvez pas vous présenter aux

visites programmées. Encore, veuillez nous informer s'il y a des changements à votre santé, même si cela ne parait pas pertinent.

- Les prothèses dentaires et les implants ont besoin de soins réguliers ; nous vous prions de bien les brosser (au moins deux fois/jour). Nous vous recommandons aussi de visiter un dentiste régulièrement après la fin de cette étude, au moins une fois/année.
- Après la fin de cette étude, vous serez responsable pour les coûts de vos soins dentaires. Eventuellement, vous aurez besoin de réparations ou de remplacement du dispositif de rétention, comme c'est le cas avec des prothèses et implants dentaires. Les coûts peuvent varier parmi les cliniques différentes ; les frais peuvent être environ 100 $ pour réparer/remplacer un dispositif. D'autres réparations peuvent être ainsi plus chers ; par exemple, ajouter de l'acrylique sur la surface qui couvre la gencive (regarnissage) peut couter 394 $ ou plus (en fonction de comment cela est fait).

**AVANTAGES ASSOCIES AU PROJET DE RECHERCHE**

Il se peut que vous retiriez un bénéfice personnel de votre participation à ce projet de recherche, mais nous ne pouvons vous l'assurer. Par ailleurs, nous espérons que les résultats obtenus contribueront à l'avancement des connaissances scientifiques dans ce domaine et au développement de meilleurs traitements pour les patients.

Tous les traitements faits pour cette étude (implant, ajustement de votre prothèse et changement des attachements durant 18 mois) n'entraîneront pas de frais.

**RISQUES ASSOCIES AU PROJET DE RECHERCHE**

Les deux dispositifs à l'étude sont déjà approuvés pour l'utilisation avec les patients et vendus au Canada et aux États-Unis. Donc, les risques associés au traitement sont les mêmes que ceux des chirurgies mineurs et prothèses dentaires amovibles déjà connus.

Ainsi, si vous constatez un effet secondaire, quel qu'il soit, au cours de ce projet, vous devez immédiatement avertir le dentiste responsable de ce projet de recherche, que vous croyiez ou non que cet effet soit en lien avec l'implant ou les dispositifs. Même lorsque votre participation à ce projet sera terminée, n'hésitez pas à contacter le dentiste responsable si vous ressentez un effet secondaire qui pourrait être lié au dispositif à l'étude.

Le dentiste responsable de ce projet de recherche et les membres de son équipe de recherche répondront aux questions que vous pourriez avoir à ce sujet. De plus, à chaque visite, le dentiste responsable de ce projet de recherche et les membres de son personnel de recherche vous poseront des questions au sujet de tous les effets secondaires que vous auriez pu avoir.

**1. Risques associés au dispositif à l'étude**

Avant la mise en place des implants, on vous injectera un anesthésiant local. Il est possible quoiqu'improbable que vous ayez une réaction allergique à ce produit. Si cela se produit, on suivra des

procédures standard pouvant comprendre des antihistaminiques ou de l'adrénaline.

Vous pourriez éprouver un changement ou une perte de sensation dans vos lèvres, vos gencives ou votre menton après la mise en place des implants. Ce phénomène est généralement de nature temporaire. Une infection peut aussi se produire, auquel cas il faudra la traiter à l'aide d'antibiotiques au besoin. Vous pourrez ressentir une certaine douleur aux gencives à l'emplacement des implants, mais cet inconfort devrait être minime et disparaître après quelques jours. Vous devriez être en mesure de calmer la douleur grâce à un analgésique sans ordonnance (par exemple, aspirine, Tylenol).

**2. Risques associés aux prothèses dentaires**

Vous pourrez ressentir de la douleur à certains endroits de votre prothèse après la mise en place de l'implant. Si cela se produit, vos prothèses dentaires seront ajustées selon le besoin. Des allergies au matériaux dentaires (par exemple, le mélange acrylique utilisé pour fixer l'attachement de rétention à la prothèse dentaire) sont rares mais pas impossible.

Votre prothèse dentaire inférieure peut casser après l'insertion des connecteurs. Si cela se passe pendant l'étude, nous réparerons votre prothèse sans frais.

**3. Autres risques**

La perte d'implant est peu fréquente mais possible. Si votre implant est perdu ou ne s'ancre pas à l'os, il sera remplacé. Vous avez encore l'option de continuer à porter une prothèse dentaire conventionnelle sans implant. Vous pouvez retourner à cette prothèse si vous le souhaitez. Les implants dentaires peuvent se détacher graduellement de l'os et provoquer l'endolorissement ou de l'infection. Si cela se produit, l'implant devra être enlevé et nous devrons possiblement vous prescrire un antibiotique pour l'infection.

Les examens radiographiques bien que les autres examens n'impliquent aucun risque ni complication. Ceux-ci comprennent la collecte des données et entrevues.

## RISQUES ASSOCIES A LA GROSSESSE

Cette étude ne comporte aucun risque de ce type en raison de l'âge minimale pour participer (65 ans).

## AUTRES TRAITEMENTS POSSIBLES

Vous n'êtes pas obligé de participer à ce projet de recherche pour recevoir des soins médicaux pour votre condition. D'autres options existent comme : (1) prothèses dentaires nouvelles ou réparations (regarnissages) ; (2) la pose de deux implant ou plus dans la mâchoire inférieure ; (3) des implants dans la mâchoire supérieure. Nous vous invitons à parler au dentiste responsable de ce projet de recherche des diverses options disponibles.

**PARTICIPATION VOLONTAIRE ET DROIT DE RETRAIT**

Votre participation à ce projet de recherche est volontaire. Vous êtes donc libre de refuser d'y participer. Vous pouvez également vous retirer de ce projet à n'importe quel moment, sans avoir à donner de raisons, en informant l'équipe de recherche.

Votre décision de ne pas participer à ce projet de recherche ou de vous en retirer n'aura aucune conséquence sur la qualité des soins et des services auxquels vous avez droit ou sur votre relation avec les équipes qui les dispensent.

Le dentiste responsable de ce projet de recherche, le comité d'éthique de la recherche, l'organisme subventionnaire ou le commanditaire peuvent mettre fin à votre participation, sans votre consentement. Cela peut se produire si de nouvelles découvertes ou informations indiquent que votre participation au projet n'est plus dans votre intérêt, si vous ne respectez pas les consignes du projet de recherche ou encore s'il existe des raisons administratives d'abandonner le projet.

Cependant, avant de vous retirer de ce projet de recherche, nous vous suggérons de notifier le dentiste responsable, verbalement ou par écrit. Nous vous demandons cela en raison de votre sécurité et pour mieux analyser les résultats.

Si vous vous retirez du projet ou êtes retiré du projet, l'information et le matériel déjà recueillis dans le cadre de ce projet seront néanmoins conservés, analysés ou utilisés pour assurer l'intégrité du projet.

Toute nouvelle connaissance acquise durant le déroulement du projet qui pourrait affecter votre décision de continuer à participer à ce projet vous sera communiquée rapidement.

**CONFIDENTIALITÉ**

Durant votre participation à ce projet de recherche, le dentiste responsable de ce projet ainsi que les membres de son personnel de recherche recueilleront, dans un dossier de recherche, les renseignements vous concernant et nécessaires pour répondre aux objectifs scientifiques de ce projet de recherche.

Ces renseignements peuvent comprendre les informations contenues dans votre dossier médical, incluant votre identité, concernant votre état de santé passé et présent, vos habitudes de vie ainsi que les résultats de tous les tests, examens et procédures qui seront réalisés. Votre dossier peut aussi comprendre d'autres renseignements tels que votre nom, votre sexe, votre date de naissance et votre origine ethnique.

Les questionnaires de recherche et rayon-x seront acheminés au bureau du D^r^ de Souza et conservés pour 25 ans aux fins exclusives du présent projet puis détruits. Son bureau est situé à l'Université McGill, Faculté de médecine dentaire, 2001, avenue McGill Collège, bureau 534, H3A1G1, Montréal (QC).

Tous les renseignements recueillis demeureront confidentiels dans les limites prévues par la loi. Vous ne serez identifié que par un numéro de code. La clé du code reliant votre nom à votre dossier de recherche sera conservé par le dentiste responsable de ce projet de recherche.

Pour assurer votre sécurité, une copie du formulaire de consentement, les données sur le type de connecteur/implant utilisés et résultats des rayon X seront versés dans votre dossier médical. Par conséquent, toute personne ou compagnie à qui vous donnerez accès à votre dossier médical aura accès à ces informations.

Le dentiste responsable de ce projet de recherche fera parvenir, au commanditaire ou à ses représentants, les données codées vous concernant.

Les données de recherche codées pourront être transmises par le commanditaire à ses partenaires commerciaux. Cependant le commanditaire et ses partenaires à l'étranger sont tenus de respecter les règles de confidentialité en vigueur au Québec et au Canada, et ce, quels que soient les pays.

Ces données de recherche seront conservées pendant au moins 25 ans par le dentiste responsable/chercheur principal (D$^r$ de Souza).

Les données de recherche pourront être publiées ou faire l'objet de discussions scientifiques, mais il ne sera pas possible de vous identifier.

À des fins de surveillance, de contrôle, de protection, de sécurité et de mise en marché du médicament à l'étude, votre dossier de recherche ainsi que vos dossiers médicaux pourront être consultés par une personne mandatée par des organismes réglementaires, au Canada ou à l'étranger, tels que Santé Canada, ainsi que par des représentants du commanditaire, de l'établissement ou du comité d'éthique de la recherche. Ces personnes et ces organismes adhèrent à une politique de confidentialité.

Vous avez le droit de consulter votre dossier de recherche pour vérifier les renseignements recueillis et les faire rectifier au besoin.

Par ailleurs, l'accès à certaines informations avant la fin de l'étude pourrait impliquer que vous soyez retiré du projet afin d'en préserver l'intégrité.

**DECOUVERTES FORTUITES**

Les découvertes fortuites matérielles sont les constatations faites dans le cadre de l'étude qui pourrait avoir des répercussions importantes sur votre bien-être actuel ou futur ou celle de vos membres de famille. Une découverte fortuite constater dans le cadre de cette recherche sera communiquée à vous et à un professionnel de la santé de votre choix.

Nous vous examinerons selon les soins standards en médicine dentaire pendant la "visite 1". Cela peut révéler quelques maladies qui sont pas le sujet de cette étude. Cependant, ces maladies peuvent se montrer important pour votre bien-être. Exemples comprissent : (1) des infections buccales; (2) tumeurs ou kystes qui sont visibles seulement par rayon X. Nous vous informerons sur ces résultats

éventuels et vous référer vers le traitement adéquat si nécessaire. Nous ferons cela indépendamment de votre participation ou non à cette étude. Si nous avons une découverte fortuite pendant quelque visite, nous ferons le même.

**POSSIBILITÉ DE COMMERCIALISATION**

Les résultats de la recherche découlant notamment de votre participation à ce projet pourraient mener à la création de produits commerciaux. Cependant, vous ne pourrez en retirer aucun avantage financier.

**FINANCEMENT DU PROJET DE RECHERCHE**

Le dentiste responsable de ce projet de recherche ainsi que l'institution ont reçu un financement du commanditaire pour mener à bien ce projet de recherche.

**COMPENSATION**

En guise de compensation pour les frais encourus en raison de votre participation au projet de recherche vous recevrez un montant de 25 $ par les visites 5, 6 et 7 prévues au protocole, pour un total de 3 visites, soit un montant total de 75 $. Si vous vous retirez du projet (ou s'il est mis fin à votre participation) avant qu'il ne soit complété, la compensation sera proportionnelle à la durée de votre participation.

Également, pendant toute votre participation à l'étude, la pose de l'implant et des components vous sera offert gratuitement.

**EN CAS DE PREJUDICE**

Si vous deviez subir quelque préjudice que ce soit par suite de l'administration du médicament à l'étude ou de toute procédure reliée à ce projet de recherche, vous recevrez tous les soins et services requis par votre état de santé.

En acceptant de participer à ce projet de recherche, vous ne renoncez à aucun de vos droits et vous ne libérez pas le dentiste responsable de ce projet de recherche, le commanditaire et l'établissement de leur responsabilité civile et professionnelle.

**INSCRIPTION DU PROJET DE RECHERCHE**

Une description de cet essai clinique est disponible sur le site www.clinicaltrials.gov (projet NCT03126942). Ce site Web ne comprendra aucun renseignement permettant de vous reconnaître. Tout au plus, le site présentera un résumé des résultats de l'étude. Vous pourrez consulter ce site Web en tout temps, ou accéder https://clinicaltrials.gov/ct2/show/NCT03126942 directement.

**IDENTIFICATION DES PERSONNES-RESSOURCES**

Si vous avez des questions ou éprouvez des problèmes en lien avec le projet de recherche, ou si vous souhaitez vous en retirer, vous pouvez communiquer avec le dentiste responsable ou avec une personne de l'équipe de recherche au numéro suivant : M. Nicolas Drolet, téléphone: (514) 398-7203, ext. 0199; courriel: nicolas.drolet@mcgill.ca; ou D[r] Raphael de Souza (chercheur principal) chez Université McGill, téléphone: (514) 398-4777; courriel: raphael.desouza@mcgill.ca.

Pour toute question concernant vos droits en tant que participant à ce projet de recherche ou si vous avez des plaintes ou des commentaires à formuler, vous pouvez communiquer avec :

(1) Le Commissaire local aux plaintes et à la qualité des services de l'Hôpital General de Montréal au numéro suivant : (514) 934-1934 ext. 44285.
(2) L'Agente en éthique de l'Université McGill (M[me] Ilde Lepore) à : ilde.lepore@mcgill.ca ou au numéro de téléphone (514) 398-8302.

**SURVEILLANCE DES ASPECTS ETHIQUES DU PROJET DE RECHERCHE**

Le comité d'éthique de la recherche du Centre Universitaire de santé McGill a approuvé le projet et assurera le suivi du projet, pour les établissements du réseau de la santé et des services sociaux du Québec.

**Titre du projet de recherche :** Prothèses dentaires complètes à recouvrement retenues par un implant unique et attachements Novaloc : un essai clinique croisé à méthodes mixtes

**SIGNATURE**

***Signature du participant***

J'ai pris connaissance du formulaire d'information et de consentement. On m'a expliqué le projet de recherche et le présent formulaire d'information et de consentement. On a répondu à mes questions et on m'a laissé le temps voulu pour prendre une décision. Après réflexion, je consens à participer à ce projet de recherche aux conditions qui y sont énoncées.

J'autorise l'équipe de recherche à avoir accès à mon dossier médical.

- J'autorise le chercheur responsable de la présente recherche à communiquer avec moi afin de me demander si je suis intéressé(e) à participer à d'autres recherches.

  Oui ☐
  Non ☐

- J'autorise le chercheur responsable de la présente recherche à informer mon médecin traitant que je prends part à cette étude.

  Oui ☐ Nom et coordonnées du médecin traitant : ___________________________________
  Non ☐
  Je n'ai pas de médecin traitant / Je ne suis plus suivi par mon médecin traitant ☐

Je comprends que le chercheur responsable enverra à mon médecin traitant des informations sur ma santé si elle sera utile pour mes soins.

_____________________________________________________________________________

Nom du participant Signature Date

***Signature de la personne qui obtient le consentement***

J'ai expliqué au participant le projet de recherche et le présent formulaire d'information et de consentement et j'ai répondu aux questions qu'il m'a posées.

_____________________________________________________________________________

Nom de la personne qui obtient le consentement Signature Date

***Engagement du chercheur responsable***

Je certifie qu'on a expliqué au participant le présent formulaire d'information et de consentement, que l'on a répondu aux questions qu'il avait.

Je m'engage, avec l'équipe de recherche, à respecter ce qui a été convenu au formulaire d'information et de consentement et à en remettre une copie signée et datée au participant.

________________________________________________________________________

Nom du chercheur responsable Signature Date
