# Supplementary material for: Single-implant overdentures retained by the Novaloc attachment system: study protocol for a mixed-methods randomized cross-over trial
Source: Trials. 2018 Apr 23;19:243. doi: 10.1186/s13063-018-2606-7 (PMC5913792; doi:10.1186/s13063-018-2606-7)
Supplement: Supplementary file 3 — Ethical approval document. (PDF 766 kb) [file 13063_2018_2606_MOESM3_ESM.pdf]

McGill

Faculty of Medicine
3655 Promenade Sir William Osler #633
Montreal, QC H3G 1Y6

Faculté de médecine
3655, Promenade Sir William Osler #633
Montréal, QC H3G 1Y6

Fax/Télécopieur: (514) 398-3870
Tél/Tel: (514) 398-3124

03 April 2017

Dr. Raphael F. de Souza
Faculty of Dentistry
2001, avenue McGill-College, Room 534
Montreal QC H3A 1G1

RE: **IRB Study Number A03-M07-17A**
Single-implant overdentures retained by the Novaloc Attachment System: a mixed-methods randomized cross-over trial

Dear Dr. F. de Souza,

Thank you for responding to the IRB's correspondence concerning the 06 March 2017 full Board review of the above-referenced study.

The submitted response and revisions are acceptable. Final ethics approval for this study is provided on 03 April 2017:

- Study Protocol (IRB dated March 2017);
- English and French Information and Consent Form, Version 1: IRB dated March 2017;
- English and French Recruitment Ad;
- English and French Questionnaires: Screening Criteria (English), VAS Practice, Assessment of Prosthesis, OHIP-20E, and Cost-Analysis – surgical/prosthetic procedures and follow-up (English).

The ethics approval for this study is valid until **March 2018.** The Certificate of Ethical Acceptability is enclosed.

All research involving human subjects is required to undergo an annual ethics review as stipulated in Federal and Provincial documents guiding and regulating research involving human subjects. This annual review is scheduled according to the date of initial approval, and it is the responsibility of the investigator to submit a completed application form for Continuing Ethics Review to the IRB prior to the stop date of the study's ethics approval. A copy of the Continuing Review form is available on the IRB website at: http://www.mcgill.ca/medresearch/ethics/.

The Investigator is reminded of the requirement to report all IRB approved study documents to the Research Ethics Offices (REOs) for the participating study sites, if applicable. Please contact the individual REOs for instructions on how to proceed. Research funds may be withheld, and/or the study's data may be revoked for failing to comply with this requirement.

Any modifications or unanticipated developments that may occur to the study prior to the annual review must be reported to the IRB promptly. Study modifications cannot be implemented prior to ethics review and approval of the change.

The IRB has assigned this study the following **IRB Study Number: A03-M07-17A.** Please reference this number for all correspondence with our office.

Regards,

Serge Gauthier, MD
Chair (Interim)
Institutional Review Board

Cc: A03-M07-17A

McGill

Faculty of Medicine
3655 Promenade Sir William Osler #633
Montreal, QC H3G 1Y6

Faculté de médecine
3655, Promenade Sir William Osler #633
Montréal, QC H3G 1Y6

Fax/Télécopieur: (514) 398-3870
Tél/Tel: (514) 398-3124

CERTIFICATION OF ETHICAL ACCEPTABILITY FOR RESEARCH INVOLVING HUMAN SUBJECTS

The Faculty of Medicine Institutional Review Board (IRB) is a registered University IRB working under the published guidelines of the Tri-Council Policy Statement, in compliance with the Plan d'action ministériel en éthique de la recherche et en intégrité scientifique (MSSS, 1998), and the Food and Drugs Act (17 June 2001); and acts in accordance with the U.S. Code of Federal Regulations that govern research on human subjects. The IRB working procedures are consistent with internationally accepted principles of Good Clinical Practices.

At a full Board meeting on 6 March 2017, the Faculty of Medicine Institutional Review Board, consisting of:

Frances Aboud, PhD — Kelly Davison, PhD

Patricia Dobkin, PhD — Sylvie Lambert, PhD

Song Lingqiao, LL.M. — Kathleen Montpetit, M.Sc.

Roberta Palmour, PhD — Maida Sewitch, PhD

Margaret Swaine, B.A.

Examined the research project **A03-M07-17A** titled: *Single-implant overdentures retained by the Novaloc Attachment System: A mixed-methods randomized cross-over trial*

As proposed by: Dr. Raphael F. de Souza (Applicant) to ____________________ (Granting Agency, if any)

And consider the experimental procedures to be acceptable on ethical grounds for research involving human subjects.

03 April 2017
Date

Chair, IRB

Dean of Faculty

Institutional Review Board Assurance Number: FWA 00004545

STUDY PROTOCOL: SINGLE-IMPLANT OVERDENTURES RETAINED BY THE NOVALOC ATTACHMENT SYSTEM: A MIXED METHODS RANDOMIZED CROSS-OVER TRIAL

DATE OF I.R.B. APPROVAL
APR 3 - 2017
Faculty of Medicine
McGill University

Principal investigator: Dr. Raphael F de Souza, DDS, MSc, PhD
Faculty of Dentistry, McGill University
2001 McGill College, suite 534, Montreal, Quebec H3A 1G1
Tel:(514) 398-4777 ext. 00052; Email: raphael.desouza@mcgill.ca

1. BACKGROUND & LITERATURE REVIEW

Complete tooth loss or edentulism is a debilitating and irreversible condition that represents the ultimate consequence of oral disease (1). Although a modest decline in the prevalence of this condition was reported for some developed countries, there are still large numbers of edentulous individuals worldwide (2). The prevalence is higher in elderly populations, and tends to remain high for several decades (3). Edentulism is associated with greater disability and earlier mortality in elders, even after adjusting for confounders such as socioeconomic status and health behavior (4). The absence of teeth also poses a major predicament for well-being, as it has considerable negative impact on quality of life. Poorer oral function is closely associated with lower self-esteem and psychosocial discomfort (5).

The major purpose of dental prostheses is to revert masticatory impairment and poorer quality of life by replacing the lost teeth. The most common prostheses for edentulism are complete dentures, which cannot completely restore lost function, e.g. chewing performance is only 30% of that for dentate individuals (6). Many complete denture wearers are functionally impaired and, consequently, have considerable psychosocial discomfort. Such issues are mostly associated with mandibular dentures, making the mandible the primary target for dental implants (7). Many clinical studies highlight that the mandibular implant-retained overdentures is a cost-effective choice for edentulous individuals, resulting in better patient satisfaction and oral health-related quality of life compared to conventional dentures (8). Since 2002, international consensus statements have recommended the use of two implants in the mandible as the standard of care for edentulism due to favorable results and low costs than most implant-based treatment methods (9, 10). However, this treatment may be unviable in some cases due anatomic or physiological conditions that are common in elders, or even be unaffordable by certain potential recipients.

The retention of a dental complete prostheses by a single implant placed in the midline has arisen as a minimal implant-based treatment modality for the edentulous mandible. This viability of such modality was unknown at the time of the first published consensus on two-implant overdentures, but later studies have showed favorable outcomes (11). Single implant overdentures present potential advantages that may lead

Version: March 2017

McGill

Faculty of Dentistry

INFORMATION AND CONSENT FORM

Single-Implant Overdentures Retained by the Novaloc Attachment System: a Mixed Methods Randomized Cross-Over Trial

Principal investigator:
Dr. Raphael F de Souza
Faculty of Dentistry, McGill University
2001 McGill College, suite 534, Montreal, Quebec H3A 1G1
Tel:(514) 398-4777; Email: raphael.desouza@mcgill.ca

DATE OF I.R.B. APPROVAL
APR 3 - 2017
Faculty of Medicine
McGill University

Research team members: Professor Jocelyne Feine, Dr. Shahrokh Esfandiari, Dr. Nicholas Makhoul, Dr. Christophe Bedos, Dr. Samer Abi Nader, and Dr. Didem Dagdeviren
Faculty of Dentistry, McGill University

1. <u>Purpose of this Consent Form</u>

We are inviting you to participate in a research study designed to assess the success of a special type of treatment using dental implants, and to compare two types of connectors used to attach your lower denture to these implants. The results of the research will provide further knowledge about how to treat elderly patients who lose teeth. This consent form aims to:

a. inform you, as completely as possible, of the nature, purpose and risks involved in the study;

b. provide you with the necessary information you may need to decide if you will participate or not, according to your personal goals;

c. help us talk with you about your disability and its treatment.

Please read this consent form carefully and ask any questions that you may have before deciding whether or not to participate in this study. The researchers are here to help you understand completely, so please feel free to ask about anything you may want to know about the study. Please take as much time as you wish and feel free to discuss this study with your family or friends before deciding. Your participation is entirely voluntary, and if you decide not to participate, there will be no penalties or loss of benefits to which you are entitled.

If you prefer conventional dentures or are fully satisfied with your present dentures, you should NOT consider participating in this study.

Version March 2017

McGill
Faculté de médecine dentaire

FORMULAIRE D'INFORMATION ET DE CONSENTEMENT

Prothèses dentaires complètes à recouvrement retenues par un implant unique et attachements Novaloc : un essai clinique croisé à méthodes mixtes.

DATE OF I.R.B. APPROVAL
APR 3 - 2017
Faculty of Medicine
McGill University

Chercheur principal :
Dr Raphael F de Souza, D.D.S., M.Sc., Ph. D.
Faculté de médecine dentaire, Université McGill
2001, avenue McGill College, bureau 534, Montréal (Québec) H3A 1G1
Tél :(514) 398-4777; Courriel : raphael.desouza@mcgill.ca

Membres de l'équipe de recherche : Dr Jocelyne Feine, Dr Shahrokh Esfandiari, Dr Nicholas Makhoul, Dr Christophe Bedos, Dr Samer Abi Nader, et Dr Didem Dagdeviren
Faculté de médecine dentaire, Université McGill

1. **Objectifs de ce formulaire de consentement**

Nous vous invitons à participer à ce projet de recherche dont le but sera de déterminer la réussite d'une méthode de traitement avec des implants dentaires, et comparer deux connecteurs utilisés sur des implants pour maintenir en place les prothèses dentaires complètes. Les informations obtenues seront utiles pour mieux soigner les personnes âgées qui n'ont plus de dents. Ce formulaire sert à :

a. Vous informer, de façon aussi exhaustive que possible, de la nature et de l'objectif de l'étude ainsi que des risques inhérents à ladite étude;
b. Vous fournir tous les renseignements dont vous avez besoin pour décider si vous souhaitez participer ou non à l'étude, selon vos objectifs personnels;
c. Vous expliquez votre condition de santé buccale et son traitement.

Veuillez lire ce formulaire de consentement attentivement et poser toute question que vous jugez nécessaire avant de décider si vous souhaitez ou non prendre part à cette étude. Les chercheurs sont ici pour vous aider à comprendre le processus à fond et n'hésitez donc pas à leur demander quoi que ce soit relativement à l'étude. Veuillez prendre tout le temps dont vous avez besoin et n'hésitez pas à discuter de cette étude avec votre famille ou vos amis avant de prendre votre décision. Votre participation à l'étude est entièrement volontaire et, si vous décidez de ne pas y prendre part, vous ne ferez l'objet d'aucune

McGill
Faculté de médecine dentaire

DATE OF I.R.B. APPROVAL
APR 3 - 2017
Faculty of Medicine McGill University

Participant(s) needed

DO YOU WANT MORE STABLE DENTURES?

Research Project: Single-Implant Overdentures Retained by the Novaloc Attachment System: a Mixed Methods Randomized Cross-Over Trial.

Our research group at McGill University is starting a research project to **study the tightness of lower dentures retained by a single dental implant**. We will compare two types of connectors used on implants to hold dentures tight.

Results will show if a new connector works well when used on a single implant. This type of treatment can reduce costs of dentures retained by implants.

You may be eligible for this study if you:

- Are aged 65 years or over;
- Have no natural teeth for at least 6 months;
- Have a complete set of dentures in good condition;
- Are in relatively good health and able to clean your dentures.

You may not participate if you received certain types of medical care, smoke more than 10 cigarettes/day or already have dental implants. We will also use x-rays to confirm if your lower jawbone can receive an implant as planned.

If you prefer conventional dentures or are fully satisfied with your present dentures, you should NOT consider participating in this study.

Your participation will involve dental treatment and at least 3 follow-up appointments over a period of 18 months.

If you are interested in knowing more about our study, please leave your name and telephone number on the following voice mail: (514) 398-7203, ext 0199, or by email: nicolas.drolet@mcgill.ca

Principal investigator: Dr Raphael de Souza

McGill

Faculté de médecine dentaire

DATE OF I.R.B.
APPROVAL
APR 3 - 2017
Faculty of Medicine
McGill University

Participant(e)s recherché(e)s

VOULEZ-VOUS DES PROTHÈSES DENTAIRES PLUS STABLES?

Projet de recherche: Prothèses dentaires complètes à recouvrement retenues par un implant unique et attachements Novaloc : un essai clinique croisé à méthodes mixtes.

Notre groupe de chercheurs de l'Université McGill débute un projet pour **étudier la stabilité des prothèses dentaires inférieures sur un seul implant**. Nous allons comparer deux types de connecteurs sur implants pour maintenir en place les prothèses dentaires.

Les résultats aideront vérifier si un nouveau type de connecteur fonctionne bien sur un seul implant. Ce traitement peut réduire le coût des prothèses dentaires sur implants.

Vous pouvez être admissible à participer à cette étude si vous :

- Êtes âgés de 65 ans ou plus;
- N'avez aucune dent naturelle en bouche depuis au moins 6 mois;
- Portez des prothèses dentaires complètes en bonne état;
- Êtes relativement en bonne santé et en mesure de nettoyer vos prothèses dentaires.

Vous ne pouvez pas participer si vous avez reçu certains types de soins médicaux, fumez plus que dix cigarettes par jour ou avez déjà implants dentaires. Radiographies seront utilisées pour confirmer si nous pouvons poser un implant à la mâchoire inférieure comme prévu.

Vous ne devriez PAS prendre part à cette étude si vous préférez porter des prothèses traditionnelles ou êtes tout à fait satisfait de vos prothèses actuelles.

Cette étude comprendra des traitements et des suivis d'un minimum de 3 visites étalées sur 18 mois.

Si vous êtes intéressé(e)s à participer à notre étude, laissez-nous vos coordonnées sur la boîte vocale suivante : (514) 398-7203, ext 0199, ou bien par courriel : nicolas.drolet@mcgill.ca

Chercheur principal : Dr Raphael de Souza

ENTRAÎNEMENT AUX ÉCHELLES VAS

Date : ☐☐☐☐ / ☐☐ / ☐☐
a a m m j j

Code d'identification : ☐☐☐☐☐☐

Nous aimerions savoir si vous comprenez bien comment répondre aux questionnaires à l'aide d'échelles visuelles analogues. Placez un trait vertical sur la ligne horizontale à l'endroit qui représente le mieux le nombre à gauche comme dans l'exemple qui suit :

Exemple :
50% 0 ______|______ 100

**25%**	0	____________________	100	☐☐☐
**80%**	0	____________________	100	☐☐☐
**10%**	0	____________________	100	☐☐☐
**45%**	0	____________________	100	☐☐☐
**75%**	0	____________________	100	☐☐☐

DATE OF I.R.B. APPROVAL
APR 3 - 2017
Faculty of Medicine
McGill University

© McGill University, 2003

OHIP-20E Questionaire

Identification code : [| | | | | | | | |]

Date : [| | |] / [|] / [|]
a a m m j j

This questionnaire was designed to evaluate how your oral condition has affected your quality of life **during the past month**. For each of the following questions, mark the response that you feel is the best. If a question does not apply to your situation, then please indicate this just below the question.

	**In the last month:**	**Always**	**Most of the time**	**Some of the time**	**Occasionally**	**Rarely**	**Never**
1	Have you had difficulty chewing any foods because of problems with your teeth, mouth or dentures?	O 1	O 2	O 3	O 4	O 5	O 6
2	Have you had food catching in your teeth or dentures?	O 1	O 2	O 3	O 4	O 5	O 6
3	Have you felt that your dentures have not been fitting properly?	O 1	O 2	O 3	O 4	O 5	O 6
4	Have you had painful aching in your mouth?	O 1	O 2	O 3	O 4	O 5	O 6
5	Have you found it uncomfortable to eat any foods because of problems with your teeth, mouth or dentures?	O 1	O 2	O 3	O 4	O 5	O 6
6	Have you had sore spots in your mouth?	O 1	O 2	O 3	O 4	O 5	O 6
7	Have you had uncomfortable dentures?	O 1	O 2	O 3	O 4	O 5	O 6
8	Have you been worried by dental problems?	O 1	O 2	O 3	O 4	O 5	O 6
9	Have you been self conscious because of problems with your teeth, mouth or dentures?	O 1	O 2	O 3	O 4	O 5	O 6
10	Have you had to avoid eating some foods because of problems with your teeth, mouth or dentures?	O 1	O 2	O 3	O 4	O 5	O 6
11	Has your diet been unsatisfactory because of problems with your teeth, mouth or dentures?	O 1	O 2	O 3	O 4	O 5	O 6
12	Have you been unable to eat with your dentures because of problems with them?	O 1	O 2	O 3	O 4	O 5	O 6
13	Have you had to interrupt meals because of problems with your teeth, mouth or dentures?	O 1	O 2	O 3	O 4	O 5	O 6

DATE OF I.R.B.
APPROVAL
APR 3 - 2017
Faculty of Medicine
McGill University

© McGill University, 2003

QUESTIONNAIRE OHIP-20E

DATE OF I.R.B. APPROVAL
APR 3 - 2017
Faculty of Medicine
McGill University

Code d'identification : ☐☐☐☐☐☐☐☐☐☐

Date : ☐☐☐☐ / ☐☐ / ☐☐
a a m m j j

Ce questionnaire vise à évaluer combien votre condition buccale a affecté votre vie quotidienne au cours du dernier mois. À chacune des questions suivantes, cochez la case qui correspond le mieux à votre sentiment.

	Au cours du dernier mois:	Toujours	Très souvent	Souvent	Occasion-nellement	Rarement	Jamais
1	Avez-vous éprouvé de la difficulté à mastiquer des aliments à cause de problèmes dus à votre dentition, à l'état de votre bouche ou à vos prothèses ?	O 1	O 2	O 3	O 4	O 5	O 6
2	Les aliments sont-ils restés coincés entre vos dents ou dans vos prothèses ?	O 1	O 2	O 3	O 4	O 5	O 6
3	Avez-vous eu l'impression que vos prothèses étaient mal ajustées ?	O 1	O 2	O 3	O 4	O 5	O 6
4	Avez-vous eu de la douleur au niveau de la bouche ?	O 1	O 2	O 3	O 4	O 5	O 6
5	Avez-vous éprouvé de la difficulté à consommer certains types d'aliments à cause de problèmes dus à votre dentition, à l'état de votre bouche ou à vos prothèses ?	O 1	O 2	O 3	O 4	O 5	O 6
6	Avez-vous remarqué des points sensibles dans votre bouche ?	O 1	O 2	O 3	O 4	O 5	O 6
7	Vos prothèses ont-elles été inconfortables ?	O 1	O 2	O 3	O 4	O 5	O 6
8	Vous êtes-vous fait du souci à cause de problèmes buccaux ?	O 1	O 2	O 3	O 4	O 5	O 6
9	Vous êtes-vous senti(e) mal à l'aise à cause de problèmes dus à votre dentition, à l'état de votre bouche ou à vos prothèses ?	O 1	O 2	O 3	O 4	O 5	O 6
10	Avez-vous évité de consommer certains types d'aliments à cause de problèmes dus à votre dentition, à l'état de votre bouche ou à vos prothèses ?	O 1	O 2	O 3	O 4	O 5	O 6
11	Votre alimentation vous a-t-elle semblé insatisfaisante à cause de problèmes dus à votre dentition, à l'état de votre bouche ou à vos prothèses ?	O 1	O 2	O 3	O 4	O 5	O 6

© McGill University, 2003

McGill University - Faculty of Dentistry
Research Protocol: Single-implant overdentures retained by the Novaloc attachment system: a mixed methods randomized cross-over trial

Participant Code ID:

(5.A) Cost analysis – sugical/prosthetic procedures

Attachment Type: O NL O LA

[ ][ ][ ][ ] / [ ][ ] / [ ][ ]
year mm dd

Stage: Pre-load O 3 mo O 6 mo O 18 mo O Other (specify): ________

Performed procedures:	

CLINICAL TIME:

	Start	End
Application of consent form:		
Operator:		
Assistant*:		
Laboratory*:		
Others (specify:________________)†:		

*If n/a, mark "0" (zero) or cross-out; †Count any outcome data assessment done on baseline here.

MATERIAL*:

DATE OF IRB APPROVAL
APR 3 - 2017
Faculty of Medicine McGill University

Equipment:
- O Clinical exam instruments
- O Attachment kit
- O Surgical Handpiece
- O Standard handpiece and burs
- O Surgical instruments
- O Surgical kit - implants
- O Prosthetic kit – implants
- O Digital radiograph (n, 'PA':___ or 'Pan':___)
- O Laboratory bench lathe
- O Scaling instruments
- O Others:________________

	Type	Brand name/Specific type	Quantity
Consumables:	O Cotton		
	O Gauze		
	O Suturing		
	O Scalpel blade		
	O Osteotomy drills, implant		
	O Relining resin		
	O Attachment components		
	O Other (specify):		

Medication (pills or mouthwash):	

*If none, mark "0"(zero) or cross-out.

INDIRECT COSTS - PATIENT:

	Start	End
Time (since waiting in the waiting room):		

Transport Expenses

Method	Time (arrival / departure)	Cost of tickets or trip (if applicable)	Lenght (km, towns, what patient remembers)
O Walking	/		
O City bus, van or metro	/		
O Intercity bus or van	/		
O Car (self-owned or lift)	/		
O Taxi or Uber	/		
O Train			
O Others: ____________	/		

Version: February 2017

Participant Code ID:

(5.B) Cost analysis – follow-up

Attachment Type: O NL O LA

☐☐☐☐ / ☐☐ / ☐☐
year mm dd

Stage: Pre-load O 3 mo O 6 mo O 18 mo O Other (specify): ____________

Is this a scheduled visit? O Yes O No

DATE OF I.R.B. APPROVAL
APR 3 - 2017
Faculty of Medicine
McGill University

Reason of the visit:	
Procedures:	

CLINICAL TIME:

	Start	End
Operator:		
Assistant*:		
Laboratory*:		
Others (specify:____________)†:		

*If n/a, mark "0"(zero) or cross-out; †Count any outcome data assessment here.

MATERIAL*:

Equipment:	O Clinical exam instruments<br>O Attachment kit<br>O Standard handpiece and burs<br>O Surgical instruments<br>O Prosthetic kit – implants<br>O Digital radiograph (n, 'PA':___ or 'Pan':___)	O Laboratory bench lathe<br>O Scaling instruments<br>O Others:____________

	Type	Brand name/Specific type	Quantity
Consumables:	O Cotton		
	O Gauze		
	O Relining resin		
	O Attachment components		
	O Other (specify):		
Medication (pills or mouthwash):			

*If none, mark "0"(zero) or cross-out.

INDIRECT COSTS - PATIENT:

	Start	End
Time (since waiting in the waiting room):		

Transport Expenses

Method	*Time (arrival / departure)*	Cost of tickets or trip (if applicable)	Lenght (km, towns, what patient remembers)
O Walking	/		
O City bus, van or metro	/		
O Intercity bus or van	/		
O Car (self-owned or lift)	/		
O Taxi or Uber	/		
O Train			
O Others: ____________	/		

Raphael Freitas de Souza, Dr

From: Nicholas Maroun Makhoul, Dr
Sent: December-21-17 10:32 AM
To: Raphael Freitas de Souza, Dr
Subject: Fwd: Nagano Daily Activity Report / Rapport journalier d'activités Nagano

Hi Raphael,

Everything is set on our end, we should start looking at dates early in the new year.

thanks

Nick

Nicholas M. Makhoul DMD. MD. FRCD(C). Dip. ABOMS. FACS.
Maxillofacial Surgeon
Maxillofacial Oncology and Reconstructive Surgery

McGill University/Université McGill
Director and Associate Professor/Directeur et Professeur Associé
Oral and Maxillofacial Surgery/Chirurgie Buccale et Maxillo-faciale

McGill University Health Centre/Centre Universitaire de Santé McGill
Chief, Department of Dentistry and Oral and Maxillofacial Surgery/
Chef, Département de Dentisterie et de Chirurgie Buccale et Maxillo-Faciale

B3-149, 1650 avenue Cedar, Montreal General Hospital
Montreal, QC, H3G-1A4
Tel: (514) 934-1934 ext: 42492
Fax: (514) 934-8340
nicholas.makhoul@mcgill.ca
www.mcgill.ca/omfs

Begin forwarded message:

From: Nagano MUHC <donotreply.nagano@muhc.mcgill.ca>
Subject: Nagano Daily Activity Report / Rapport journalier d'activités Nagano
Date: December 21, 2017 at 2:32:19 AM GMT-5
To: <nicholas.makhoul@mcgill.ca>
Reply-To: <donotreply.nagano@muhc.mcgill.ca>

***** La version française suit *****

Hello Dr Nicholas Makhoul,

This is your Nagano-MUHC daily activity report for 20 December 2017.

Activities related to your projects were issued. Some of them may require an action from you, while others may have already been processed.

3 activity(ies) key(s) to your projects:

- The form **F20 - 23811** for the project **2018-3873: Single implant overdenture** is: ***Approved***
- The project **2018-3873: Single implant overdenture** is: ***Authorized for research***
- The form **F11 - 22298** for the project **2018-3873: Single implant overdenture** is: ***Approved***

Only activities that you have requested are sent. If you do not wish to receive these notices, go to you your profile tab followed by the "Activities on my Projects" tab, to make the necessary changes. The profile icon is in the upper right corner of your screen. The procedure is also available in the documentation section of Nagano and on our website.

Have a good day,

The MUHC REB Team

Bonjour Dr Nicholas Makhoul,

Ceci est le compte-rendu de vos activités Nagano-MUHC pour le 20 December 2017.

Des activités liées aux différents projets que vous suivez ont été émises. Certaines d'entre elles peuvent requérir une intervention de votre part, alors que d'autres ont peut-être déjà été traitées.

3 activité(s) touche(nt) vos projets :

- The form **F20 - 23811** for the project **2018-3873: Single implant overdenture** is: ***Approved***
- The project **2018-3873: Single implant overdenture** is: ***Authorized for research***
- The form **F11 - 22298** for the project **2018-3873: Single implant overdenture** is: ***Approved***

Seules les activités que vous avez sélectionnées vous sont envoyées. Si vous désirez ne pas recevoir ces avertissements, allez dans your profile, onglet "Suivi d'activités", pour y apporter les changements nécessaires. L'icône de profil se trouve dans le coin supérieur droit de votre écran. La procédure est également disponible dans la section documentation de Nagano et sur notre site web.

Bonne journée,

L'équipe du CÉR du CUSM

Centre universitaire de santé McGill | McGill University Health Centre

2017-10-12

Dr. Nicholas Makhoul
1640 Cedar Avenue
Room B3-149.1
Montreal, Quebec
H3G1A4

email: nicholas.makhoul@mcgill.ca

Re: REB Conditional Approval of a New Research Project (Single implant overdenture / 2018-3873)

"Study Protocol: Single-Implant Overdentures Retained By The Novaloc Attachment System: A Mixed Methods Randomized Cross-Over Trial"

MUHC REB Co-Chair for the Clinical Trials 2 (CT2) Panel: Dr. Bertrand Lebouche

---

Dear Dr. Makhoul,

Thank you for the initial submission of the research project indicated above.

The McGill University Health Centre (MUHC) Research Ethics Board (REB), more precisely its CT2 Panel has reviewed the research project at its full board meeting of 2017-10-04 where quorum was reached. Please be advised that no REB member withdrew from the deliberations.

The *Initial Submission Form* (F11-22298), as well as the following documents, were reviewed:

- McGill IRB Approval Letter (2017-04-03]
- McGill Consent Form (V1 2017-04-03) in French and English
- External science review (2016-11-15)
- External science review (2017-01-19)
- Letters of collaboration / support (2016-08-15)
- Nespaper Ad (V2 2017-08-16) in French
- Recruitment Documents (V1 2017-03-31) in French
- Recruitment Brochure (V1 2017-03-31 in English
- Information & Consent Form (V1 2017-03-31) in French and English
- Research Protocol (V3 2017-09-21)
- Investigator's Brochure (V1 2010-01-01)
- Screening Questionnaires (V1 2017-03-31) in English
- Satisfaction and Rotation Questionnaire (V1 2017-03-31) in French and English
- OHIP Questionnaires (V1 2017-03-31) in French and English
- Economic Data Collection Form (Undated)
- Cost Analysis, PO and Follow Up (V1 2017-03-31)
- McGill IRB Initial Review Form (V1 2017-02-10)

- Cover Letter/Summary (V1 2017-02-10)
- Approval of the Department / Division Head (2017-09-26)

After reviewing the documents, **this research project was approved unanimously by the MUHC REB conditional upon the receipt of responses to the conditions listed in the *REB Conditions & PI Responses Form* (F20-23811) and documents attached to it.** This will be reported to the MUHC REB and will be entered accordingly into the minutes of the next CT2 Panel meeting.

Corrected documents attached to the F20-23811 will have to be submitted in “track changes”.

We trust this will prove satisfactory to you. Thank you for your consideration in this matter.

Best Regards,

MUHC REB Coordinator
for MUHC REB Co-chair mentioned above
